# From Empire-wide integration to regional localization: A synthetic and quantitative study of heterogeneous amphora data in Roman Germania reveals centuries-long change in regional patterns of production and consumption

**DOI:** 10.1371/journal.pone.0279382

**Published:** 2023-01-11

**Authors:** Tyler Franconi, Tom Brughmans, Ekaterina Borisova, Laura Paulsen

**Affiliations:** 1 Joukowsky Institute for Archaeology & the Ancient World, Brown University, Providence, Rhode Island, United States of America; 2 Classical Archaeology, Aarhus University, Aarhus, Denmark; 3 Centre for Urban Network Evolutions, Aarhus University, Aarhus, Denmark; 4 Center for Humanities Computing Aarhus, Aarhus University, Aarhus, Denmark; Universita degli Studi di Foggia, ITALY

## Abstract

We present novel insights into trade in amphorae-borne products over a 550-year period in Germania along the frontier of the Roman Empire, derived through probabilistic aoristic methods to study temporal changes in archaeological materials. Our data analysis reveals highly detailed differential patterns of consumption and production within the German market. We show how connections to far-flung regions such as the Eastern Mediterranean or the Iberian Peninsula wax and wane through time, and how the local German producers start to compete with these imported products. These chronological patterns provide important insight into a regional market within the larger Roman economy and provide an important case study in changing economic connections over a long period, demonstrating in a transparent and reproducible way a geographical and chronological pulsation in market activity that was otherwise unknown and undemonstrated.

## Introduction

It is now widely accepted that many commodities moved over long distances throughout the Roman Empire (ca. 30 BC—AD 500), ranging from basic foodstuffs like grain to expensive luxuries such as ivory [1,2]. Efforts to understand and quantify this movement by archaeologists and historians have substantially refined our understanding of the origins, transportation, and consumption of these goods [1,3–5], but are often hindered by the imperfections inherent to archaeological data. Different research agendas, catalog typologies, and quantification methods each contribute to the creation of heterogeneous data that present numerous difficulties in understanding the spatial and chronological extent of Roman trade and, by extension, its wider economic context. These obstacles are not insurmountable, and we prefer to see archaeological data as characterful rather than obstructive [6,7], thus highlighting their strengths while acknowledging and accounting for weaknesses. There is a need to use quantitative methods that allow for explicitly identification of the impacts of data heterogeneity if we are to obtain a better understanding of how long-distance movements of foodstuffs occurred throughout the Roman Empire and changed over centuries, and if we are to leverage the potential of excavated and published material culture from large numbers of Roman sites. In this paper we present results of such a quantitative exploration using probabilistic aoristic methods: an approach that is much needed but exceedingly rare in Classical Archaeology as it is able to account for much of the chronological ambiguity present in these data [8–10].

**Fig 1:**
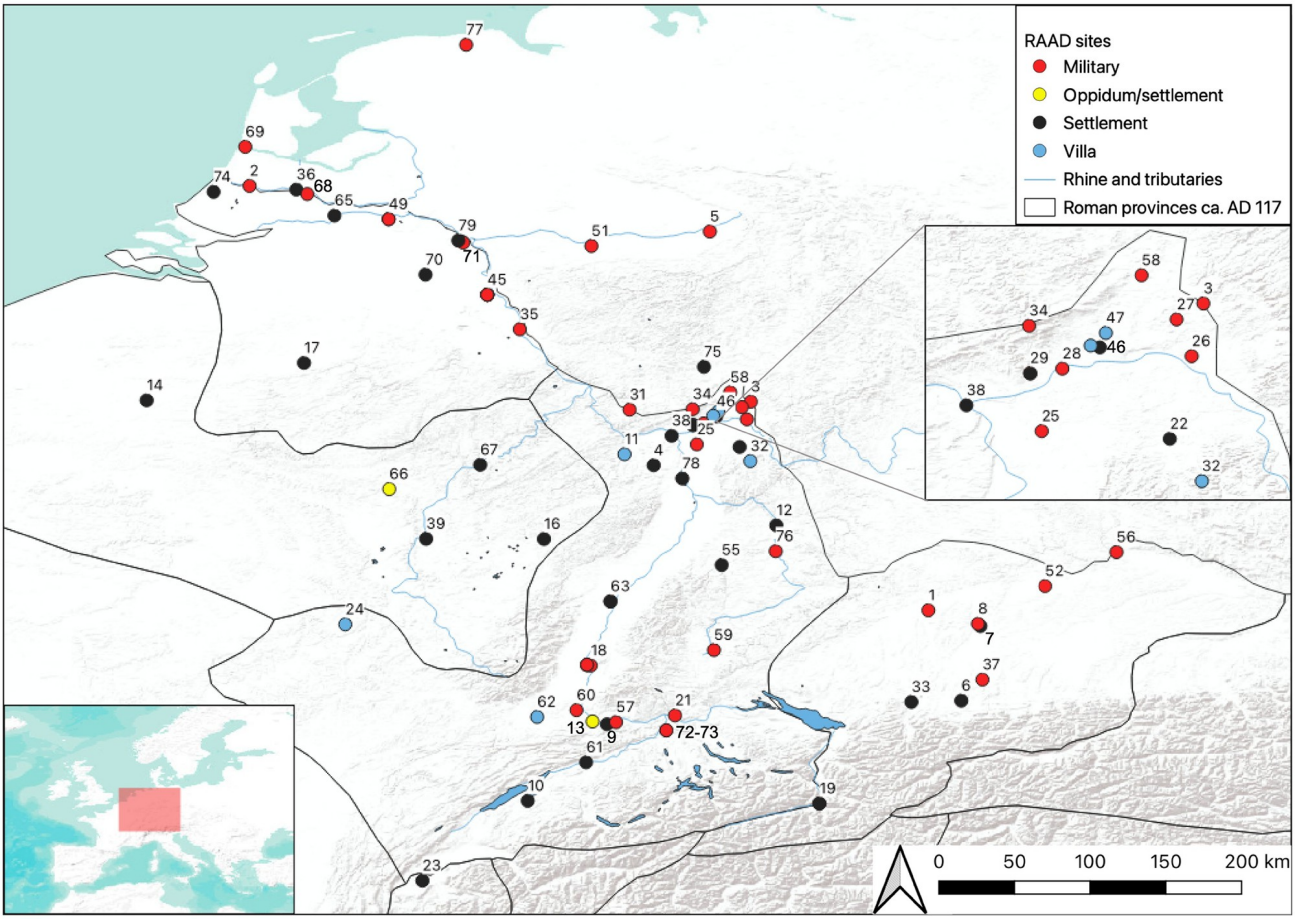
Map of RAAD sites. Sites colored by site type, with inset detailing the region of northern Hessen. Labels correspond to RAAD site number.

We demonstrate this for the case of Roman trade as evidenced by large ceramic transport vessels known as amphorae from the region of ancient Roman Germany, roughly equivalent with the parts of The Netherlands, Germany, France, and Switzerland that border the Rhine River in western Europe (Fig 1). This region is a prime case study for three reasons: first, these countries have over 150 years of archaeological research that has been published in high detail with a tradition that especially emphasizes the importance of artefactual study. Second, the region was a key frontier zone of the Roman Empire and, despite its peripheral geographical location, the German frontier was often central to Roman history. Finally, as a frontier region, Roman Germany also allows for a particular view of economic development that is geographically removed from the Mediterranean core. Much of Roman economic history has focused on the Mediterranean exclusively, and frontier zones have rarely featured in any detailed discussion, despite their rich archaeological datasets. In fact, many prevailing models of Roman economic development see Roman frontiers as economically insignificant regions that depended almost entirely on State subsidized redistribution networks that first prioritized the supply of frontier armies with products [11–14]. These models ignore the economic agency of provincial civilian populations that, especially after the first century AD, were far more numerous than the soldiers stationed within them [15]. These models also fail to account for geographical and chronological differences, and instead see all frontier regions as static through time. Amphorae, as vessels that mainly carried foodstuffs, offer an important insight into this economic activity, as they relate not only to long-distance trade, but also to production and marketing strategies at both the macro and micro scales. Thus, the region has much to offer to economic historians that are interested in exploring data-based analyses that are able to account for both chronological and geographical dynamism.

There are, however, many considerations when synthesizing data at this scale. Ceramic excavation, analysis and publication practices in this region reflect high heterogeneity of ceramic quantification methods, typologies used, temporal evidence and research agendas. For example, the site of Augusta Raurica (RAAD site 9, modern day Augst) in Basel-Landschaft, Switzerland, is perhaps the most detailed chronological study available, reflecting the high-quality of excavation, study, and publication at the site [16–18], but many sites fall short of this scale of publication because researchers are operating within challenging constraints such as using archived museum collections that are in some cases a century old [19]. These conditions of study and publication directly affect our ability to synthesize and interpret these assemblages, and they have an impact on our analyses. We explore many different facets of these influences throughout this paper and summarize the main findings below in the discussion. Despite these issues, this heterogeneous information is aggregated in the RAAD database that we analyze in this paper, which has so far assembled published amphorae assemblages from 79 different archaeological sites in this region (Fig 1), and includes over 260 individual ceramic forms [15,20,21].

This synthetic database forms a foundation for quantitative analysis of ceramic data at a geographical and chronological scale not yet otherwise achieved in Roman studies. The RAAD material allows for comprehensive analysis of the German frontier region’s consumption and, to some extent, production strategies at the regional level and allows us to move beyond site-based, anecdotal approaches that extrapolate single case studies to represent entire regions. This is achieved through the following wide range of exploratory data analysis queries. We will explore the impacts on data patterns between using production and consumption dates (respectively amphora type and site dates), and between ceramic quantification methods (Minimum Number of Individuals (MNI), Maximum Number of Individuals (MAX), and diagnostic Rim, Base, and Handles count (RBH)). We will additionally trace temporal patterns of amphorae from different regions (Iberian Peninsula, Italy, Gaul, Eastern Mediterranean, North Africa, and Germania), with different typical prime-use contents (wine, olive oil, fish sauce, fruit). Finally, we will explore differences between amphora data patterns at different site types (military, settlement, villa), explore the exceptional case of Augustan period sites with a short occupation span, and closely examine the data pattern of the site of Augst that has a particularly rich, published material record.

The regional, long-term, and replicable quantitative view on Roman trade patterns reflected through the amphorae data analysis results presented here is novel in its scope and scale. First, a synthetic view of Roman amphora consumption in one region through time has never been attempted at this scale–instead patterns are typically inferred through anecdotal use of one or several individual sites. Second, the heterogeneity of the amphora data has hindered efforts at quantification through time, especially since the individual quantification methodologies have differed so much by publication: this paper tackles the impact of this heterogeneity head-on. Third, the centuries-long patterns evident in these data provide new and important insights into the temporal fluctuations of Roman imperial commerce differentiated into regional markets. That is, the German frontier zone is used as a case study against which we can compare wider Roman imperial patterns.

## Materials and methods

### Dataset creation

The Roman Amphorae Assemblage Database (RAAD), developed in conjunction with the Oxford Roman Economy Project’s (OXREP) aims to tackle Roman amphora data heterogeneity by taking a synthetic view of Roman trade [15,20,21]. It includes published amphora data from 79 archaeological sites in Roman Germany, with 260 individual amphora typologies recorded (Fig 1). This dataset is unique because of its synthetic nature that incorporates data from a wide range of published sources and presents them in a digital (and therefore easily analyzed) form, and there are no clear parallels from other regions in the Empire. The Southampton University Amphora Project [22] developed the most comparable dataset, though its focus was on amphora typologies across the Empire, including production origin, contents, and date range which built upon pioneering work by amphorae specialists over many decades especially in the Mediterranean (e.g. [23–26]. This resource was an invaluable building block in the creation of RAAD. We extracted the full dataset from this online resource and numbered each form from 1 to 219, corresponding to their alphabetical order in the database [21]. This extraction allowed for cross-referencing the forms found and published in Germany, and we found that 148 forms overlapped between the datasets and that RAAD added a further 112 that were either discovered since its publication, or that had been left out due to their regional specificity [17,19,27–34]. Thus, RAAD provides a substantial increase in knowledge related to typology and synthesis that builds upon both Mediterranean and Northern European research traditions and allows for quantitative data analyses that explore highly detailed amphora trade patterns through time.

**Fig 2:**
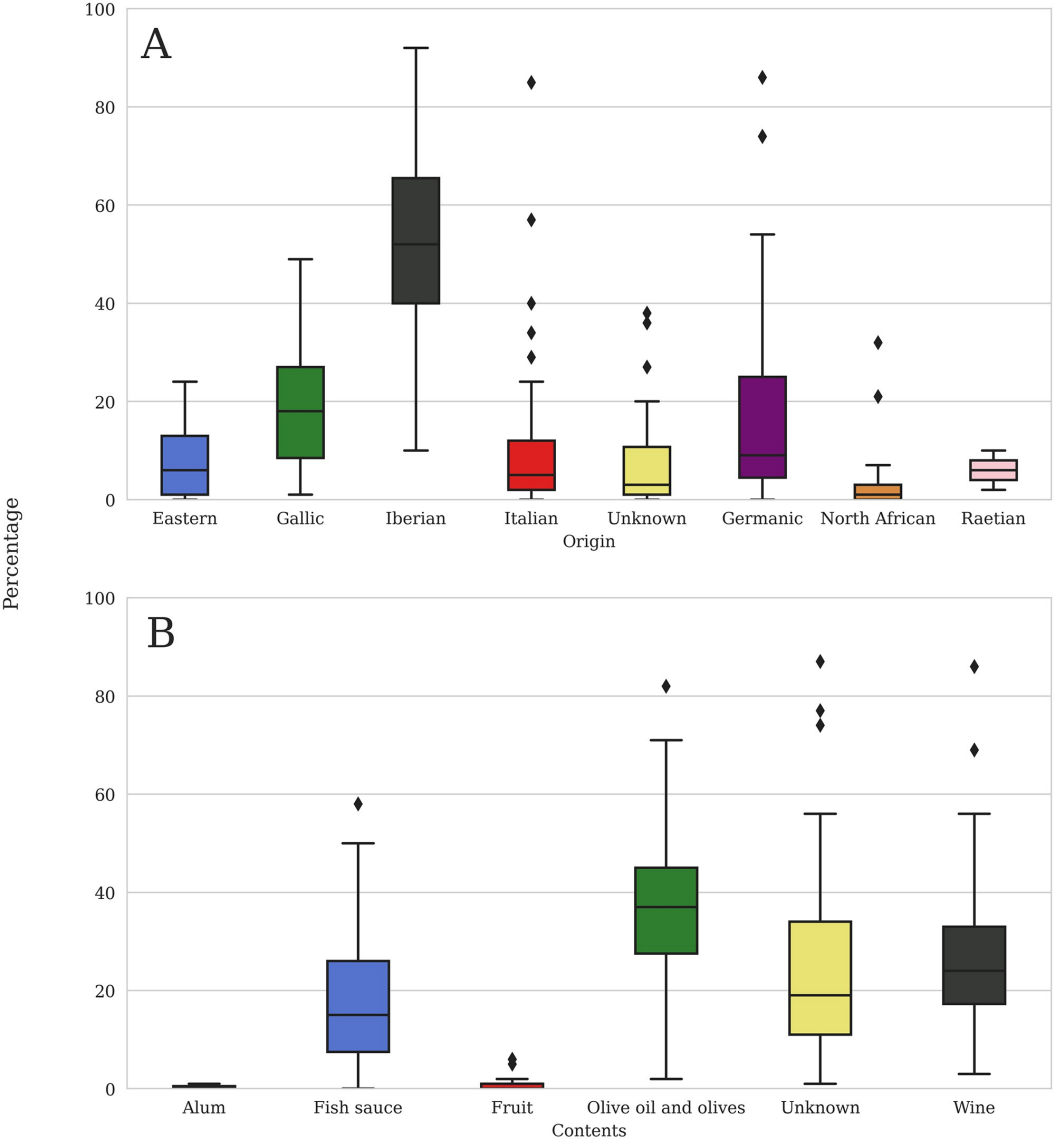
a-b. Amphora origins and contents. Box and whisker plots showing percentage of each origin and content as part of each site assemblage.

The RAAD project synthesized diverse datasets into several standardized tables that accounted for information about amphora forms as well as the archaeological sites on which they were found [21]. The “Amphora Form” identification utilizes four main columns: RAAD type number, RAAD form, Origin, and Contents. These four headings allow all amphora forms to be discussed on similar footing. The ‘RAAD type’ number assigns a specific number to each amphora form to standardize the terminology of typology. For instance, RAAD form 28 is synonymous with the previous typologies of Camulodunum 189, Kingsholm 117, Oberaden 85, Schöne Mau XV, Dressel 18, and Carrot which are used interchangeably and, for many, confusingly. The ‘RAAD form’ column contains these typological equivalences, so that a specific form can always be checked against its RAAD type number. The ‘Origin’ column indicates where the amphora was made, broadly grouped into eight regions of the Roman world: The Eastern Mediterranean (including Greece, Turkey, the Levant, and Egypt–these regions produce a very small portion of the overall dataset and therefore were amalgamated into a single region that would be visible on the figures. See [20] for more discussion), Gaul, Germania, Italy, North Africa, Raetia, the Iberian Peninsula, and Unknown. Where possible, further subregions were specified; for instance, Iberian amphorae can be further subdivided into Southern Spain, the Guadalquivir Valley, Lusitania, Marismas, and Tarraconensis. It is noteworthy that Iberian and Gallic products dominate these regional assemblages, with other regions supplying far fewer products across the timespan (Fig 2a).

The ‘Contents’ column records the most commonly carried product in each form: alum, fish sauce, fruit, olive oil, olives, wine, and unknown. While there are some hypotheses about some of the ‘unknown’ contents (such as RAAD form 105, the Dressel 20 similis which is produced throughout the Rhineland in the second and third centuries AD which may have carried vegetal oils, beer, or even dairy products [19] we have decided to leave them marked as unknown in order to reflect the ambiguity. This categorization of both origin and contents assumes that each container cataloged here was deposited in the archaeological record after its initial use. It is not within the scope of this study to perform a quantitative assessment of the reality of reuse [e.g. 35], which may have utilized a container for goods beyond its original intended prime-use content (so-called Type A reuse, [36]), given the general paucity of direct evidence for reuse. Olive oil containers form the largest percentage of site assemblages, followed by wine, and fish sauce (Fig 2b). Finally, each form is given a production ‘Start date’ and ‘End date’ derived from their standard type-chronologies, which importantly marks when the form was made rather than when it was consumed.

The table for ‘Site Data’’ contains extensive information about the archaeological sites from which the amphorae assemblages came [21]. Each assemblage is numbered and then identified by ‘modern site name’, ‘ancient site name’ (if known), the ‘modern country’, the ‘Roman province’, and then its geographical coordinates in lat/long. Each site is also linked to one or multiple online atlases such as Pleiades, the Digital Atlas of the Roman Empire, and Vici. The sites are then classified by type and sorted into four categories: military, settlement, oppidum, villa. These types reflect the character of Roman occupation–military sites include legionary fortresses, auxiliary forts, and late-Roman castella while settlements include the civilian settlements around these fortifications in addition to the numerous cities and towns throughout the region. Villas were a type of rural estate, typified by large and often lavishly decorated houses set within an agricultural landscape. Oppida were pre-Roman hillforts that were often continually used through the Roman period. There are, of course, more types of sites than these four broad categories in the study region, but these are the types that produced published amphora reports. Smaller rural sites, like farms, are largely missing from this analysis, and this is a result of scholarly practice rather than ancient reality.

Each site is also given occupation start and end dates, ranging from 100 BC at the Titelberg oppidum in Luxembourg (site 66) to AD 450 at the settlement of Mainz, Germany (site 38). Thus, the database covers some 550 years of amphora consumption in the region. In contrast to production dates noted in the Forms table, these dates indicate the chronology of consumption. Finally, each site is noted for how its ceramic data are quantified: diagnostic rims, bases, and handles (RBH); minimum number of individuals (MNI), and maximum number of individuals (MAX) as well as how large each assemblage is. The sum of amphora frequency across all 79 sites and using these different quantification methods is 33,707 and it is important to note that some of the published amphora evidence concerns only diagnostic amphora sherds rather than a total quantification. These two datapoints are perhaps the most important details in the database, as they determine which sites are directly comparable in quantification method–while it is reasonable to compare the raw assemblages of Xanten, Germany (RAAD site 75) with Nijmegen Kops Plateau (RAAD site 50) because both use MNI, it is unreasonable to compare directly the raw assemblage from Xanten directly to Dangstetten, Germany (RAAD site 21) because it uses MAX. This issue of different quantification methods is central to our improved methodologies outlined below.

The RAAD dataset is intended to act as a model for future study of other regions, and thus all of these attributes are easily replicated in other geographies. The dataset can also be extended to include other regions and standardized according to these tables. Thus, while this paper focuses on the German frontier, the data organization has a wide applicability and will facilitate future synthetic studies of amphorae.

### Data analysis method

To enable our data analysis, the original RAAD database [21] was restructured into a single dataframe and saved into a comma-separated values (CSV) file ‘RAAD_data_restructured.csv’. To that end, the pandas package was used [37,38]. This file includes a single row per site-type combination, and columns for each attribute related to that site, type and combination (i.e. each row contains all information about the quantity of a certain amphora type found at a certain site; about the type such as its production data and typical contents; and about the site such as the occupation dates and quantification method). See Table 1 for a list and description of all attributes.

**Table 1:** Attributes of restructured RAAD.CSV file used for this analysis.

Field Name	Type	Description
’RAAD_form’	string	Commonly used typologies for RAAD type number
’RAAD_type_number’	integer	RAAD unique form identifier
’origin’	string	A region where an amphora was produced
’origin_h1’	string	A region where an amphora was produced
’origin_h2’	string	A subregion of a region (where an amphora was made)
’contents’	string	A product carried in an amphora
’site_name_modern’	string	A modern name of an archaeological site from which an amphorae assemblage came
’site_name_ancient’	string	Roman name for sites (where known)
’site_number’	integer	RAAD unique assemblage identifier
’modern_country’	string	Modern national location of sites
’roman_province’	string	Ancient provincial location of sites
’major_site_type’	string	A site category: military settlement oppidum villa
’minor_site_type’	string	Specific types of sites, where available
’quantification_method’	string	The method with which each assemblage was quantified
’quantification_abbreviation’	string	A ceramic quantification method: total—total sherd count mni—minimum number of individuals rbh—diagnostic rim, base, and handles count max—maximum number of individuals
’raad_type_start_date’	float	A production start date of an amphora
’raad_type_end_date’	float	A production end date of an amphora
’site_start_date’	integer	A consumption start date of an amphora
’site_end_date’	integer	A consumption end date of an amphora
’frequency’	integer	An amphora frequency
’southampton_type_number’	float	Corresponding entry in Southampton Roman Amphorae: a Digital Resource (if available)
’pleiades’	float	Site geographical coordinates according to the Pleiades Atlas
’dare’	float	Site geographical coordinates according to the Digital Atlas of the Roman Empire
’vici’	float	Site geographical coordinates according to the Archaeological Atlas of Antiquity
’lat’	float	Site geographical coordinates in latitude
’long’	float	Site geographical coordinates in longitude
’total assemblage size’	integer	Total number of amphorae/sherds per site
’reference’	string	Bibliographic reference for assemblage publication

It is worth noting that text data such as *site* names (see the full list in Appendix A) has been pre-processed: punctuation marks were removed, double spaces were replaced by single spaces, and all characters were lowercased. The motivation behind this strategy was to avoid inconsistency in spelling caused by manually filling out the original spreadsheets, and thus to prevent mistakes in calculations.

The analysis method aimed to study changes in production and consumption of amphorae over time is based on the probabilistic aoristic approach [39–42]. This probabilistic approach is rarely used in Roman archaeology (most notably [10,43], despite having much to offer the study of Roman economic history. It was implemented by using the Python programming language using the following packages: pandas [37,38], regex [44], Beautiful Soup [45], seaborn [46], and Matplotlib [47]. The resulting pipeline is openly available in the form of IPython Notebook documents [48].

We focused our data analysis on identifying long-term change in three data patterns: (1) the frequency of amphorae in a given year, (2) the number of sites on which amphorae were found in a given year, and (3) the number of different amphora types present in the region in a given year. In accordance with the aoristic method, the probability *P*(*t*_*i*_) of amphorae being present in a given year *t*_*i*_ was calculated as,

$$P\left(t_{i}\right)=\sum_{c}\frac{F_{c}}{\Delta \tau }\;\;\text{if}\;t_{i}\in \Delta \tau ,$$ (1)

where *F*_*c*_ is the frequency of an amphora item *c* of a particular *form* and *Δτ* = *τ*_*end*_ − *τ*_*start*_ is the time range. The latter can be either a *production* date range of *c* (defined by the amphora type’s start and end dates) or a *consumption* period of an amphora (defined by *site* start and end dates). The summation runs over all *c* in case *t*_*i*_ belongs to the interval *Δτ*.

Each amphora item *c* was found at a particular *site* and has a specific *type number*. Thus, in addition to the calculation described above for amphora frequency (data pattern 1), the count of unique *sites* at which *c* was present in a given *t*_*i*_ (data pattern 2) as well as the count of unique *type numbers* per year (data pattern 3) were computed. The mentioned values were obtained by (i) forming a set of all unique *sites*/*type numbers*, for which *t*_*i*_ ∈ *Δτ* and (ii) counting the number of elements in it.

Much like the dataset creation, the data analysis method is intended to have a broad applicability outside this case study, and likewise can be used to expand this study into other regions of the Roman Empire where chronological uncertainty is equally problematic.

### Open data and method

The original RAAD database is openly available online [21]. A Github archive is openly available [48] including the ‘RAAD_data_restructured.csv’ data file, python scripts implementing the algorithms used, documentation, and iPython notebooks implementing all results obtained. Each notebook concerns one figure, allowing for efficient evaluation, reproduction and modification of the long-term ceramic data patterns published in each figure.

## Results

Our analysis reveals important patterns of trade in amphorae, chronological trends in the consumption of goods depending on both place of origin as well as contents. These chronological trends in turn reflect changing tastes among consumers as well as changing market connections. Both are significant for understanding trade at the local, regional, and Empire-wide level in the Roman Empire. In this section we describe the data patterns, and we interpret them in full detail in their historical and archaeological research contexts in the subsequent discussion section.

### Consumption vs. Production chronologies

Chronological trends differ, in some cases substantially, when dates are analyzed by the production chronologies of individual amphora forms (type dates) or by the chronologies of the sites on which they are consumed (site dates). Neither method necessarily fully captures the complete reality of ancient trade, but both contribute different aspects to our analyses when viewed side by side. A huge spike in amphora frequency around the turn of the era is only visible when using site dates (Fig 2a), and clearly reflects an extreme example of the fact that sites were occupied for different lengths of time: the spike is created by Augustan period sites that were occupied for a very short period (see below). For instance, the site of Anreppen (RAAD site #5), a military base along the Lippe River in northern Germany, was occupied only between AD 4–9 and this five-year period therefore reflects its consumption chronology. During this time, the soldiers stationed at the site consumed wine from the Eastern Mediterranean in containers such as Dressel 2-5s (RAAD type 23) or Camulodunum 184/Rhodian amphorae (RAAD type 31) that were produced over much longer spans of 300 and 175 years respectively. These extensive production chronologies obscure the very short occupation of the site, and make it seem as if the chronology of importation is much longer than it actually was. This is an extreme example but makes the point clearly: production and consumption chronologies are not the same. Despite these clear disparities, a combination of both methods in each of our analyses can be used to reveal different patterns depending on the question asked. It must be noted that amphorae with ‘unknown forms’ (i.e., RAAD types 261–268) have consumption dates based on site chronologies, but do not otherwise have known production chronologies. Thus, in the discussion that follows, their numbers are added into “site date” chronologies, but not “type date”. These are very small quantities of material (a total frequency of 1236), and so do not skew these figures in any meaningful way, but their presence is worth noting.

**Fig 3:**
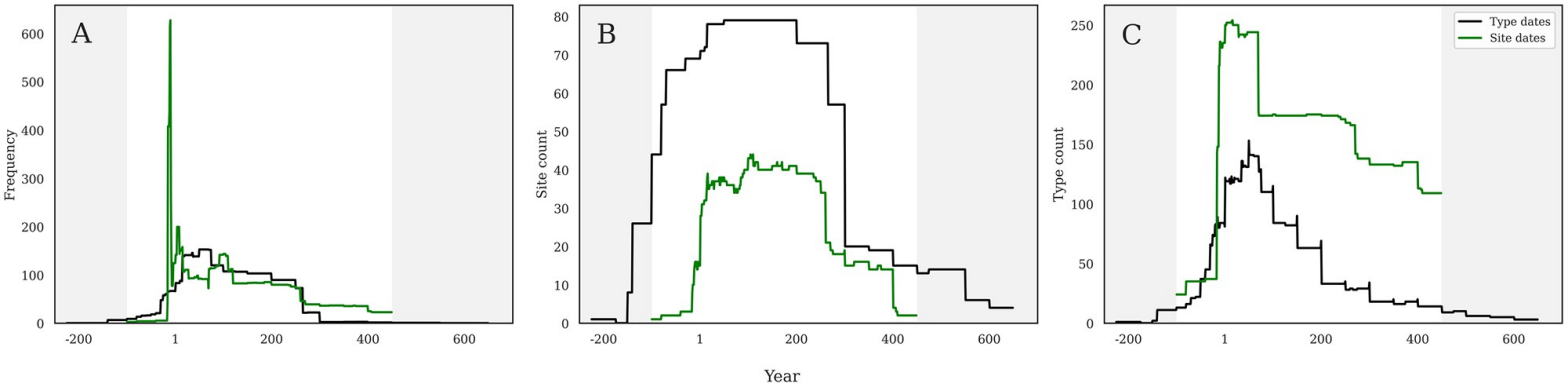
a-c. Chronological methods. Comparison of type date (green) and site date (black) chronologies across the three different methods of analysis. Note that Y axes are not constant.

Fig 3a shows the comparison between the two dating methods when considering sum of frequency for the entire dataset. Using production chronologies, we see an even chronological distribution, beginning in the late first century BC, climbing to an early first century AD peak, and then essentially disappearing by AD 300. When consumption dates are used, though, we see a very different pattern: an incredibly sharp peak just before AD 1, followed by much lower levels until ca. AD 260 that then slowly decline to ca. AD 450.

Fig 3b shows the comparison between the two dating methods when considering the count of sites, where both dating methods show a similar shape, though with some key differences. Production chronologies make the curve start extremely early, ca. 150 BC, which is 50 to 70 years earlier than the earliest sites in the dataset: Titelberg (RAAD site 66) ca. 100 BC and Basel Münsterhugel (RAAD site 13) ca. 80 BC. This method reaches its peak by about AD 1, and then plateaus for nearly two centuries, before beginning to drop after AD 200, and then sharply at AD 300, before stepping down even past AD 600. The consumption date chronology is much more constrained, only beginning ca. 100 BC, with a sharp increase just before AD 1, and then a less consistent plateau until about AD 260, before dropping to lower levels by AD 300 and essentially disappearing after AD 400. In this case, the site-based chronology is much more reflective of an ancient reality than the type-based chronology.

Fig 3c shows the comparison between the two dating methods when considering the diversity of forms in RAAD type count, and here again we see substantially different shapes through time. Type dates create a stepped pyramid starting ca. 150 BC, peaking ca. AD 75, and then steadily declining until AD 600. This pattern is again outside the actual chronological bounds of the dataset and predates the earliest site occupation by about 50 years and reaches beyond the end of Roman rule by more than a century. The site-based chronologies, however, show a start ca. 100 BC, a very sharp rise ca. AD 1, a brief plateau until AD 75 that then drops to a lower level until ca. AD 260, and then an abrupt drop followed by a slow reduction until ca. AD 450.

It must be emphasized that the type dates span a much longer period than the actual dataset, while the site dates are by definition tightly constrained within a historical reality. The 79 sites in the database fall between roughly 100 BC and AD 450, while the x-axes on these graphs range from 200 BC to AD 650. The plots of frequency generally respect the database timespan, though the type dates grow both earlier and later when plotted by site count and type count. This is again due to the broad production chronologies of some forms that are produced either earlier or later than the Roman occupation of Germany. It is clear, however, that most of the data from any of these figures falls within the correct date range, so even cutting out these temporally extraneous sections before 100 BC and after AD 450 does not change our results in any meaningful way.

### Original quantification methods

There are numerous ways to quantify ceramic assemblages, but this dataset is mainly composed of three different approaches: calculating a minimum number of individuals (MNI), counting the total sherds and hypothesizing a maximum number of individuals (MAX), or counting the main diagnostic sherds of rims, bases, and handles (RBH). Most modern studies will produce tables that contain all this information, plus weight and estimated vessel equivalents (i.e., Remesal-Rodriguez 2019), but this is not always the case when material comes from curated collections or older excavations. It is worth noting that the MAX quantification method is used exclusively by Ulrike Ehmig, and thus the shape of these data is directly formed by the sites she has researched and published, i.e., Dangstetten (RAAD site 21), Mainz (RAAD site 38), and the central German region generally. Because these different methods count different things, they are not directly comparable in raw terms; thus, a site with 12 MNI is not the same as a site with 12 MAX or 12 RBH, and indeed may represent very different quantities of material. With this in mind, we split the dataset into sites by each quantification method in order to compare the influence that quantification methodology has on the data and graphed each according to each of our three methods by both dating chronologies (production and consumption).

**Fig 4:**
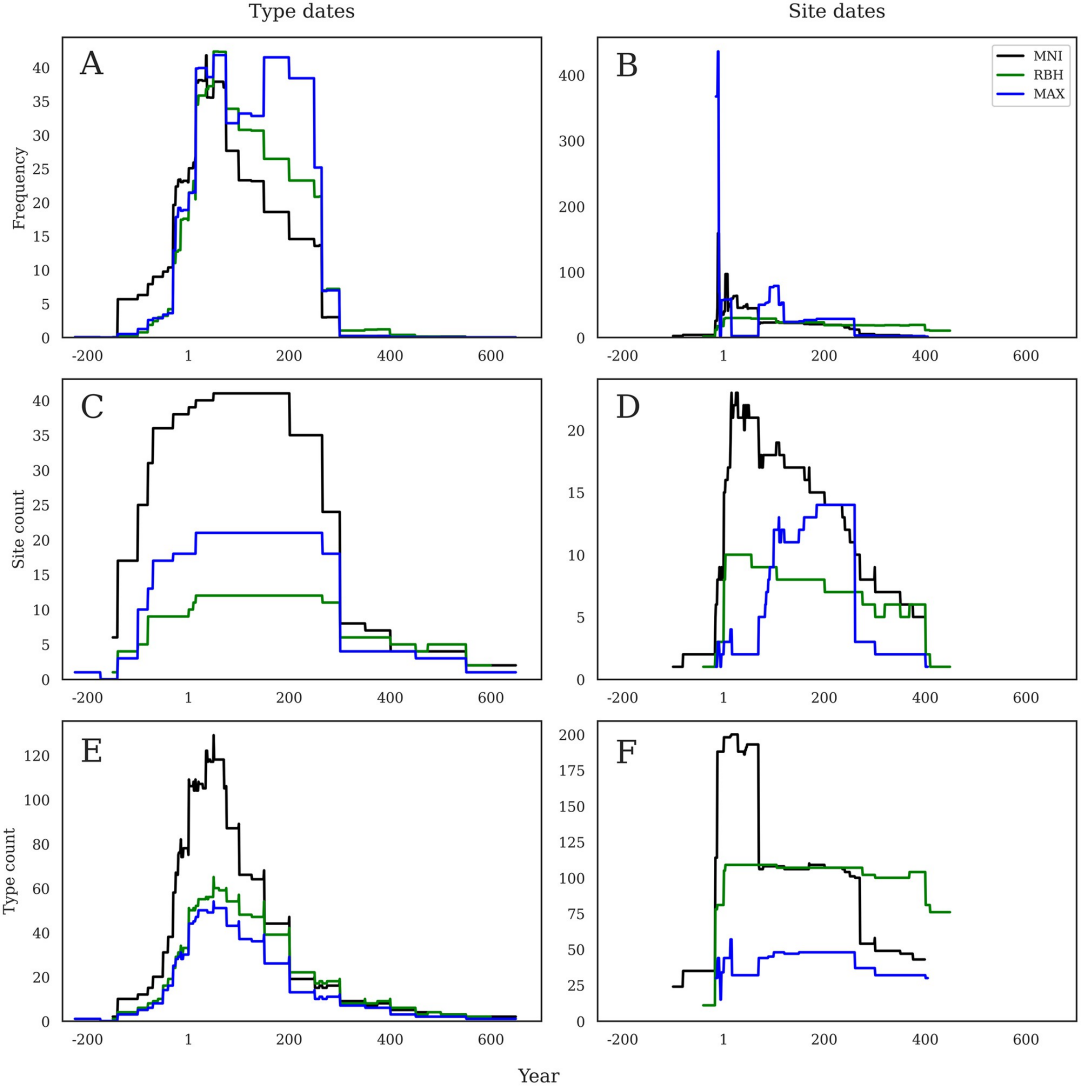
a-f. Comparison of quantification methods. Graphs show comparison across the three different methods of analysis according to production dates (left) and consumption dates (right). Note that Y axes are not constant.

When considering the sum of frequency by production dates (Fig 4a), we see marked differences in chronology between the different quantification methods. Sites using MNI show a peak in the middle of the first century AD, sites using RBH show a peak slightly later, towards the end of the century, and sites using MAX peak just before AD 100. Sites with MNI and RBH then decline in stepped form until about AD 260, but MAX reaches a second lower peak in the second half of the second century AD. When the same method is visualized using consumption dates (Fig 4b), there is an incredibly sharp peak in the last decades BC on sites with MAX quantification (reflecting the Augustan period sites quantified using this method), while the others are much more evenly spread out across the time span.

When considering the sites per year by production dates (Fig 4c), all three quantification methods show a very similar shape through time, though with MNI now dominating the count of sites quantified with this method. In contrast to the sum of frequency, all three lines show a similar rise in the first century BC, a plateau from about AD 50–300, and then lower quantities through the end of the Roman period. When considered by consumption dates (Fig 4d), however, the similarities in chronological patterning disappear; instead, we see MNI peak early in the first century AD, RBH have a flat pattern that steps down gradually from the start of the first century AD, and then MAX not even really rising until after AD 100, and then peaking in the first half of the third century.

Finally, when considering amphorae type count by production dates (Fig 4e), we again see a similar shape in all three lines: a sharp rise from the last quarter of the first century BC with a peak in the middle of the first century AD, followed then by a stepped decline until about AD 300. Sites using MNI show the highest numbers, and MAX the lowest. These similarities again disappear when considered by site dates (Fig 4f), and here we see a sharp rise in MNI followed by a sharp decline by AD 100, a very flat RBH line from ca. AD 1–400, and then an early prominence of MAX ca. AD 1 and then a plateau from ca. AD 100–260.

### Changes in regional product consumption

**Fig 5:**
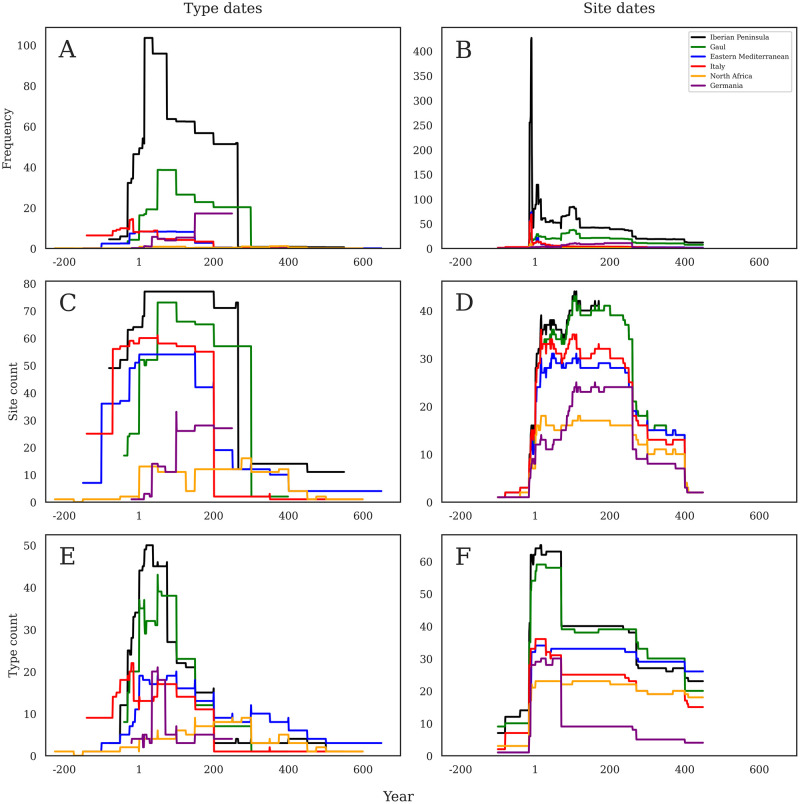
a-f: Amphora origins through time. Comparison of six main amphorae production regions across the three different methods of analysis according to production dates (left) and consumption dates (right). Note that Y axes are not constant.

**Fig 6:**
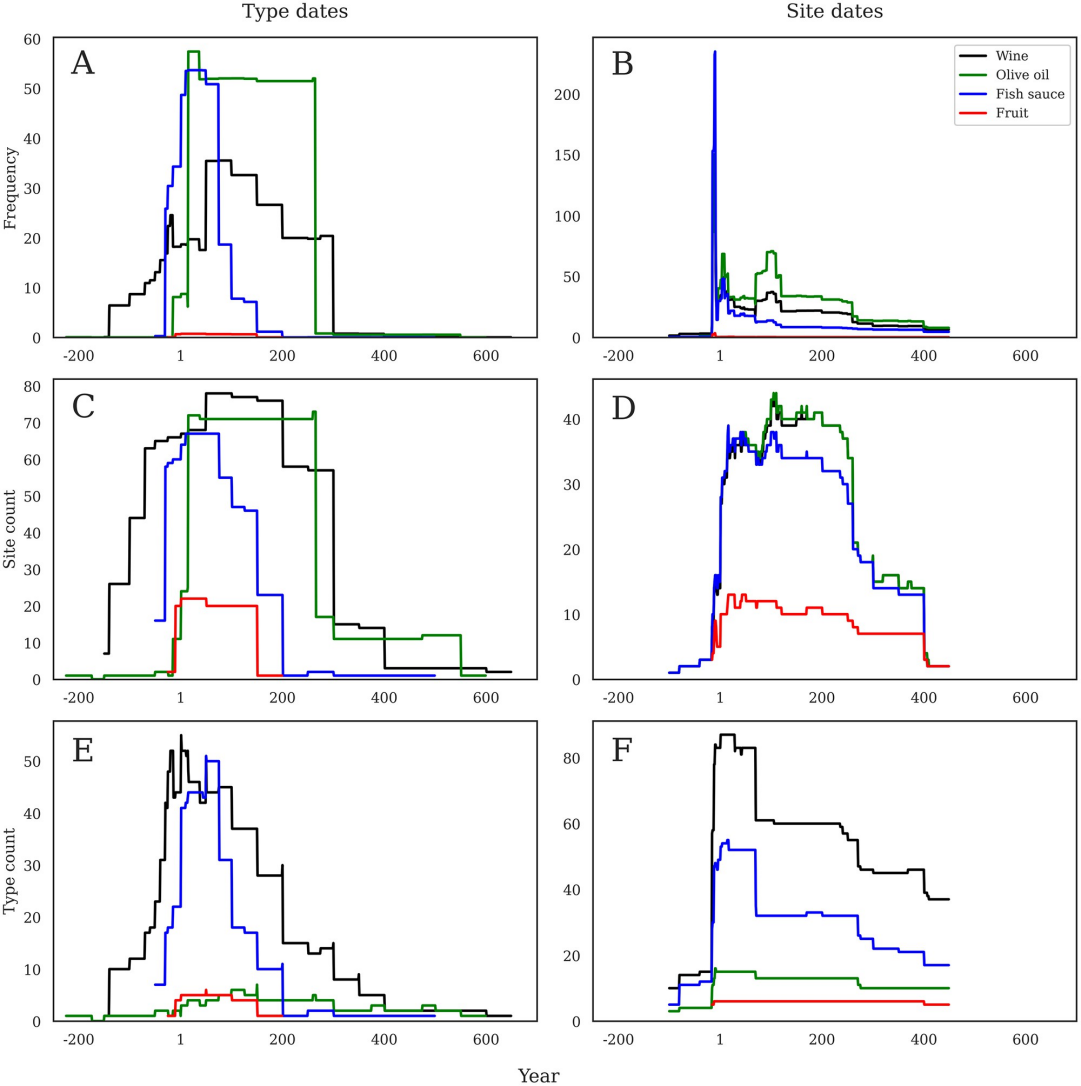
a-f. Amphora contents through time. Comparison of six main amphora-borne foodstuffs across the three different methods of analysis according to production dates (left) and consumption dates (right). Note that Y axes are not constant.

The total dataset can be broken down into two major avenues of investigation: product origin (Fig 5) and product contents (Fig 6). Fig 5a demonstrates a dominant frequency of products from the Iberian Peninsula, with a rapid rise at the end of the first century BC, a peak in the early first century AD, and then a significant decline ca. AD 75, followed by a slowed, stepped descent until ca. AD 260 when these products then plummet. Gallic products are the second most obvious in this visualization but show a pattern of a slower growth beginning in the early first century AD that then peaks ca. AD 100, before slowly dropping and declining until about AD 300, when they disappear. Compared to the Iberian and Gallic products, those from the Eastern Mediterranean, North Africa, and Italy barely feature on this figure. The sudden arrival of German products ca. AD 150 is significant and approaches the same frequency as Gallic products at the same period. However, while Gallic products drop and then disappear, Germanic products simply cease.

These figures change dramatically when visualized using consumption chronologies. Fig 5b shows a sharp and dramatic peak in the late first century BC, especially in Iberian products, that then drops quickly into the start of the first century AD (caused by the Augustan period sites). Iberian products are still most common here, and Gallic still in second place, though the other production regions are difficult to spot.

When looking at the count of sites using production chronologies (Fig 5c), the Iberian and Gallic materials maintain their dominant positions, but are more closely approached by Italian and Eastern Mediterranean products. The rapid declines of all these products in the third and fourth centuries is noteworthy, while the very low count of sites with African products stays relatively steady throughout the period, even outlasting those more common containers from western Europe.

Fig 5d uses production chronologies to visualize site count per origin and shows a much more nuanced chronology of all regions. Iberian and Gallic material is much more evenly paced, and the distinctions between the products seen in other visualizations are less apparent. These regions share a fast increase in the number of sites at which they appear at the start of the first century AD and a fast decrease ca. AD 260. The other regions are clearly less common in the dataset still, but again are shown more evenly at a large number of sites, especially Italian and Eastern Mediterranean products. The German material is even more apparent now from ca. AD 100–260, and the North African material holds steady over the course of four centuries.

Fig 5e shows the number of RAAD forms per year dated by their production chronologies. Here, the Iberian and Gallic dominance in the first two centuries AD is most clearly seen, both showing rapid rises from the late first century BC, a peak in the second half of the first century AD, and then steep declines by AD 200. Italian and Eastern Mediterranean material shows a similar pattern, but with much smaller quantities. North Africa shows a different pattern, with a slow rise over the first two centuries AD, and a peak in forms by about AD 250. Eastern Mediterranean forms are also more numerous in the fourth and fifth centuries than other regions. Here we must remember that this is only showing the number of different RAAD forms present, not the actual quantity of amphorae. Thus, a higher peak means a larger number of forms, but not necessarily a higher number of amphorae.

Fig 5f shows the number of RAAD forms per year dated by their consumption chronologies. The difference between this figure and Fig 5e is substantial, though it still shows the immediate dominance of Gallic and Iberian forms in the first century. The rest, though, shows a much flatter pattern across forms from all regions. Quantities differ, but the chronological variation is less pronounced. Gallic and Eastern forms remain most numerous after AD 100, followed by Iberian, Italian, and North African. The later North African peak shown in Fig 5e is not apparent.

### Changes in product content consumption

The shapes of these visualizations are broadly similar when compared to those of the types of products carried within the amphorae. The frequency of contents by production chronology (Fig 6a) shows a much earlier start for wine importation than other products, starting ca. 150 BC and gradually growing to a peak ca. AD 75–100. Fish sauce is much more constrained. With a rapid rise in the late first century BC and a rapid descent by about AD 100. Olive oil shows a different shape, resembling more of an olive-oil plateau that begins at the start of the first century AD and then ends ca. AD 260. The other contents barely register, reflecting their low frequency.

When visualized by consumption date, we again have substantial changes in chronological patterning. Fig 6b shows the frequency per content by consumption dates, and still shows that same sharp late first century BC spike caused by the Augustan period sites, here shared by wine, fish sauce, and olive oil. Fish sauce is notably high early on, and olive oil only becomes the dominant product towards the end of the first century AD. The earlier start date of wine importation is still visible but insignificant, and wine maintains a secondary position until about AD 260.

Turning to site counts per product contents (Fig 6c), the same trends are apparent, though in differing quantities. Wine still starts early, but now reaches well into the fourth century, and it appears on more sites than olive oil. Fish sauce still rises quickly and then falls fast as well, but now stretched over a longer period of the first two centuries AD. The olive oil mesa is still apparent from the early first to the middle of the third centuries AD. Fruit also now registers, but only at a small number of sites in the first ca. 150 years of Roman occupation.

When visualized by site count per product contents using consumption chronologies (Fig 6d), we again see a much more similar simultaneous rise in wine, olive oil, and fish sauce from the late first century BC to the early first century AD, and these products are present on a similar number of sites throughout the first century AD. Around AD 100, fish sauce becomes less common (though does not drop precipitously until ca. AD 300), though wine and oil are roughly equal throughout the Roman period, sharing a similar drop with fish sauce ca. AD 260 and then being present on a similar quantity of sites until AD 400.

Fig 6e shows the number of RAAD forms dated by their production chronologies, and especially emphasizes the long-standing diversity in amphorae forms that contained wine. Wine containers form a stepped pyramid beginning ca. 150 BC, peaking in the early first century AD, and then ending ca. AD 400. Amphorae containing fish sauce are the next most diverse category, with a tighter chronology from the late first century BC, peaking by about AD 100, and then disappearing by AD 200. Containers for olive oil show a remarkable lack of diversity, and this low number of forms is consistent from ca. AD 1 until the late third century AD. A small number of fruit containers are contained within the first century and a half AD.

Fig 6f again emphasizes the wide variety of both wine and fish sauce containers, but now dated by site chronologies. The number of different wine containers jumps in the late first century AD, and then declines again ca. AD 100, before leveling out to an even plateau for the next three centuries. Fish sauce containers show a similar pattern, though with lower numbers. Olive oil is slightly more apparent here than in the last figure, also jumping ca. AD 1 and then remaining fairly even throughout the rest of the Roman period. Fruit containers show an almost flat line throughout the entirety.

### Case study 1: Wine preferences by region

**Fig 7:**
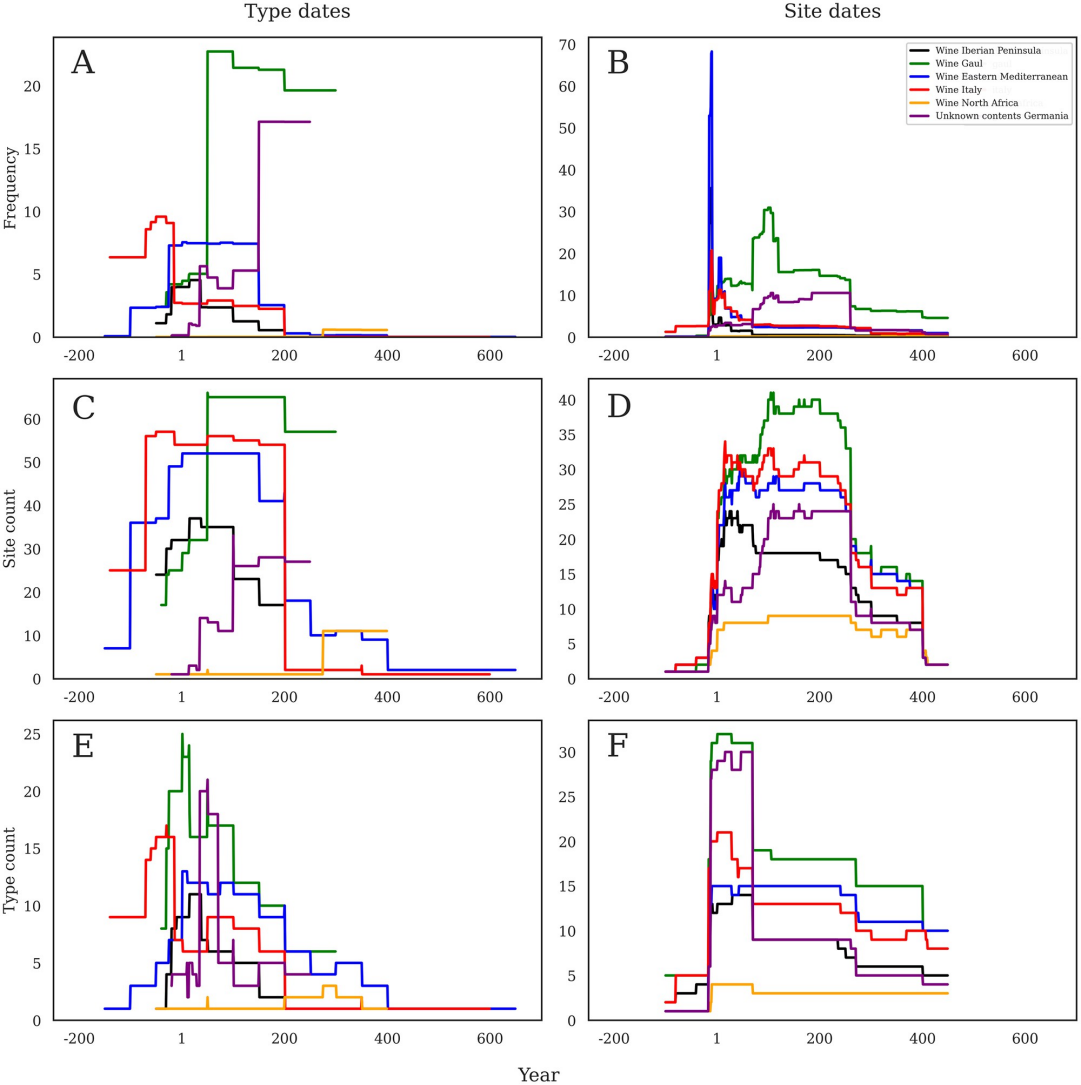
a-f. Wine consumption through time. Comparison of six main wine-producing regions across the three different methods of analysis according to production dates (left) and consumption dates (right). Note that Y axes are not constant.

This macro-view of amphora contents in the German region also allows for a more detailed view of specific products to better understand how things like wine and oil were consumed. Fig 7a shows the summed frequency according to production dates and presents an extremely heterogeneous pattern within this discrete part of the dataset. Italian wine shows an early prominence that already declines by the end of the first century BC, and then maintains a low level through about AD 200. Eastern Mediterranean wine shows a roughly inverse pattern at the start, with low levels present before the late first century BC, but then jumping to a higher level until ca. AD 150, before then declining rapidly. Iberian wine is less common, and most present in the early first century AD before dwindling to basically nothing by AD 200. Gallic wine stands out as the clearly dominant product in the region, with a very sharp rise ca. AD 50, and then high levels until ca. AD 200. We also included amphorae produced in Germany whose contents are unknown but may well have contained wine (especially RAAD form 106, the Niederbieber 74/75) or at least another alcoholic beverage like beer. When these forms are included here, they form an interesting pattern against the other regions, showing a sudden rise ca. AD 150 and then achieving a high plateau (though still below Gallic wine), until they abruptly disappear by AD 260.

Fig 7b shows the summed frequency dated by consumption chronologies, and thus a very different pattern. The sharp peak in the late first century BC shows a hierarchy of Eastern, Iberian, and then Italian wine in this period, but incredibly short lived in each case due to the short occupation span of the Augustan period sites. Gallic wine really takes off ca. AD 75, and peaks around AD 100, before declining slightly but maintaining dominance throughout the rest of the Roman period. The German amphorae with unknown contents appear here ca. AD 75 and rise to the second highest frequency until ca. AD 260 when they disappear.

Fig 7c with the number of sites with wine containers dated by production dates, again showing the widely consumed Italian and Eastern Mediterranean wines at an early date. Here, though, they last until ca. AD 200. Iberian material again shows a first century AD peak, and then disappears by AD 200. Gallic material rises sharply in the number of sites on which they are attested in the middle of the first century AD and then becomes the most widely available wine until about AD 300. The German vessels appear in the first century AD, but reach their highest levels in the second century, before disappearing ca. AD 260. North African wine only appears briefly ca. AD 300. It is noteworthy here that very few sites have wine containers in the later Roman periods.

Fig 7d shows the number of sites with wine containers dated by consumption dates. Here, the shape of each region’s curve is similar, but the quantities are vastly different. The late first century BC peak disappears, and instead we see a rapid rise in Italian, Eastern Mediterranean, Iberian, and Gallic material, that then level out into different popularities: Gallic wine at the top, then Italian, then Eastern until ca. AD 260. Iberian wine has a brief spot of popularity in the first century AD, but then is replaced by German amphorae ca. AD 100. North African wines remain rare from AD 1 to 400.

Fig 7e examines the number of different wine amphora forms present in the database according to production chronology. Here we see an early start to both Eastern Mediterranean and Italian vessels, with Italian wine amphorae forms peaking in diversity in the middle of the first century BC before steadily declining towards AD 200, when they virtually disappeared. The Eastern material shows a different pattern, where a slower (but still early) start that peaks around the start of the first century AD, but then maintains a relatively steady presence of different forms through the end of the second century, with fewer thereafter. Eastern material is present until the beginning of the 5th century AD and maintains a higher form diversity in the later period than any other region. A wide variety of Gallic wine amphorae arrive rapidly from the end of the first century BC and peak in number of forms during the first century AD. Their diversity then declines, but they maintain a high degree of diversity in vessels until the end of the second century AD. Iberian wine peaks in diversity in the early first century AD, and then gradually steps down to a very low diversity of wine amphorae in the later second century. North African wine amphorae are never very diverse, and only a handful of forms are present over the duration of Roman rule. The locally produced German material shows a different pattern, with a rapid increase in form production in the early first century AD, peaking in the middle of the century, and then reducing rapidly into the second before disappearing in the third.

Fig 7f shows a substantially different pattern in amphora form diversity when considered by site chronology rather than production. Here, we see all regions sharing a first century AD peak, with Gallic and German amphorae showing the highest degree of diversity, followed by Italian, Eastern, and Iberian wine containers. North African products are still the least diverse. This early peak then drops to a series of long-lasting plateaus, wherein Gallic material maintains the highest degree of diversity, followed by Eastern, Italian, German, and Iberian forms, with Africa still the lowest.

### Case study 2: Olive oil preferences by region

**Fig 8:**
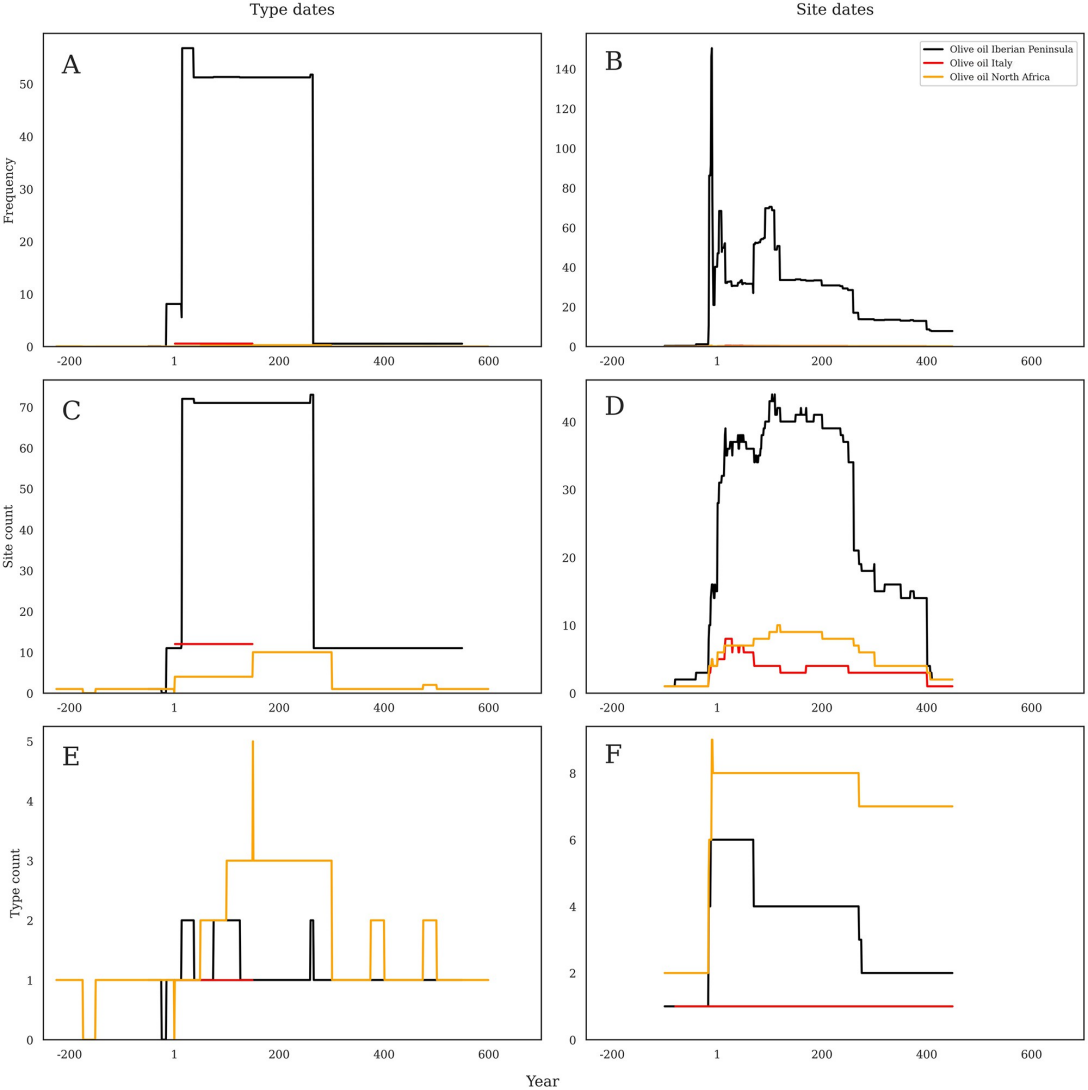
a-f. Olive oil consumption through time. Comparison of three main olive oil-producing regions across the three different methods of analysis according to production dates (left) and consumption dates (right). Note that Y axes are not constant.

Where wine was a dominant and sought-after commodity from many parts of the Roman Empire through time, olive oil shows a very different pattern. Fig 8a–8f above showed a high popularity of olive oil containers in summed frequency calculations as well as site count per year. However, there were very few different forms of olive oil containers, and this lack of diversity resulted in very low counts of RAAD types per year. It is worth digging into this patterning a bit more to understand better what the realities of olive oil trade and consumption were like in this region. It is important to note that only three oil-producing regions are present in the dataset: the Iberian Peninsula, Italy (especially Istria), and North Africa and thus not all regions are present in the following analyses.

Fig 8a shows the summed frequency distribution of oil by region dated by production dates, and the dominance of Iberian, but especially Baetican, olive oil is striking. A sharp rise just after AD 1 that leads to a dominant plateau until ca. AD 260. The other regions barely even feature in this figure, and basically no olive oil is present after the middle of the third century AD.

Fig 8b shows summed frequency by consumption dates, and still shows an Iberian dominance, but now with much more chronological nuance. The late first century BC spike is evident, followed by other peaks in the early first century AD and another ca. AD 100, and then a longer presence through to about just after AD 400. Again, the Italian and North African material is barely visible.

Fig 8c shows site count per year by production dates and is very similar to Fig 7a in that it shows a dominant Iberian oil market from ca. AD 1 until 260. Here, though, we can see the signs of other regions, with an early Italian presence from ca. AD 1 until 150, and then a slightly later North African presence, especially from about AD 150 until 300.

Fig 8d shows site count per year by consumption date and adds some nuance to the pattern otherwise seen in Fig 7c. Iberian oil is still dominant, with a sharp rise ca. AD 1, and then a sustained presence until AD 260, but then a still notable presence until AD 400. The other regions are also visible. With an early Italian peak ca. AD 25–50, and then North African material becoming more present after about AD 100 and remaining visible until after AD 400.

Fig 8e considers the diversity of olive oil amphorae present in the database according to production chronology. Here, North African oil shows the earliest diversity, but with only two forms. Italian oil is equally early, but with only one. Iberian material starts similarly to Italy, but then increases at the very end of the first century BC to a high of six forms. This then drops by the end of the century to a more limited repertoire over the rest of the Roman period. North African material, interestingly, remains highly heterogeneous across the Roman period in Germany, maintaining a high degree of form diversity from the start of the first century AD through the end of the fifth.

Fig 8f shows the diversity of olive oil amphorae present in the database according to site occupation chronology, and the pattern is again very differently shaped from the production chronology. North African oil starts notably earlier (the earliest date is due to the identification of a Maña C1 (RAAD form 179) at Dangstetten, Germany (RAAD site 21), see (49), but then disappears for a brief period before oil again reappears in the late first century BC. North African amphorae then gradually diversify up to a mid-second century AD peak, and then drops again to only one or two forms after AD 300. Iberian material arrives from the late first century BC and reaches its most diverse point in the first and second centuries AD with a maximum of two different forms that then last through the end of the Roman period. Italian material arrived at the same time but only briefly in more than one vessel in the first century AD.

### Case study 3: Augst, Switzerland

**Fig 9:**
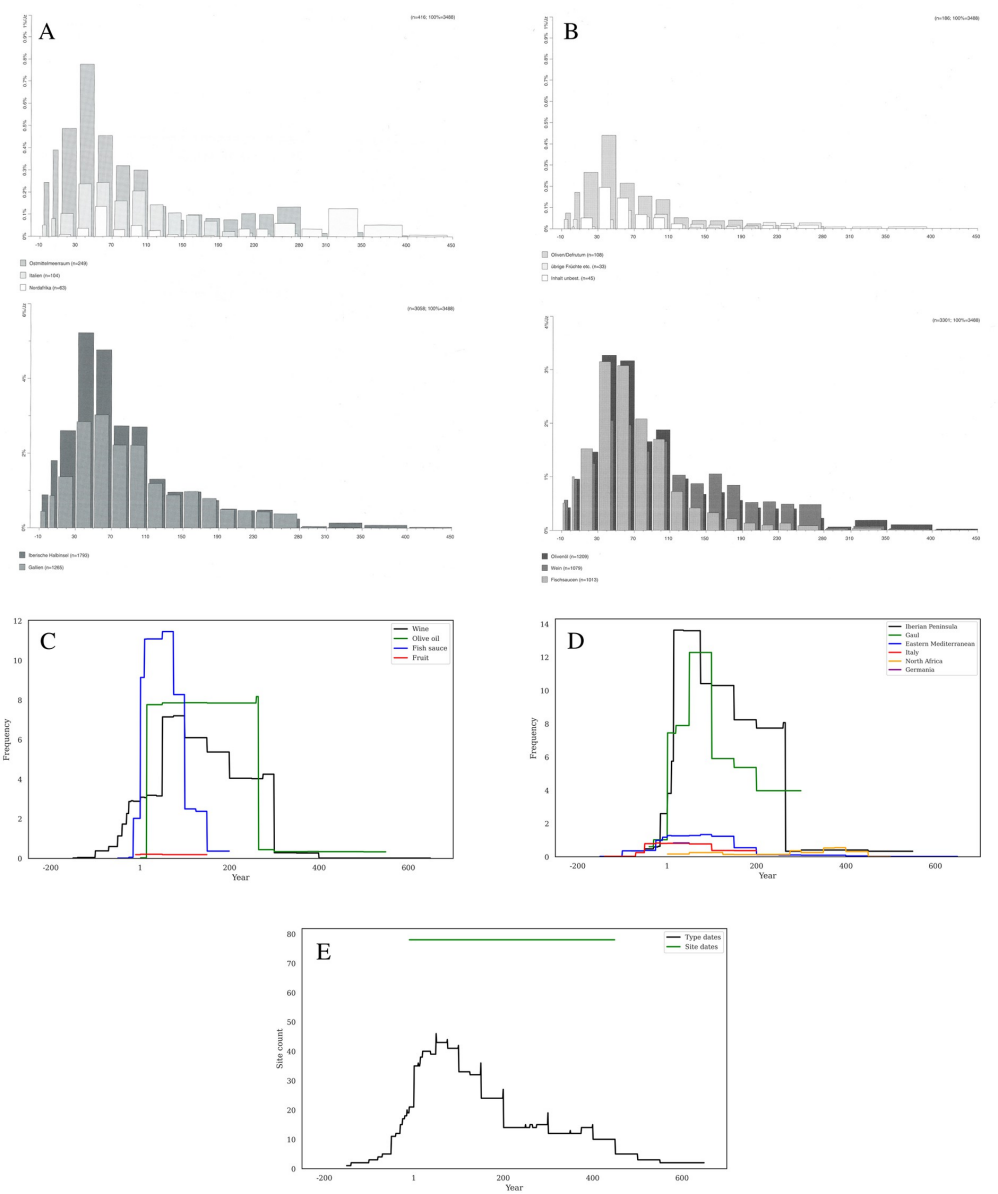
a-e. Amphorae consumption at Augst, Switzerland. Comparison of phased amphorae consumption data from Augst, Switzerland (Martin-Kilcher 1994 Figs 208 and 210 by contents (a) and origin (b)), with our analyses by origin (c), contents (d), and form diversity (e). Note that Y axes are not constant.

All of these investigations have focused on the German frontier at the macro-scale, presenting the full synthesis of data from 79 sites within the region. It is important to also understand how individual sites fit into this regional pattern, and so we also compare our methods of analysis with the well-studied and highly-resolved dataset from Augst, Switzerland (RAAD site 9) [16–18]. The original publication of these amphorae presented a chronology of consumption at the site over the course of some 460 years, from 10 BC to AD 450 (Fig 9a and 9b) that utilized the site stratigraphy and artefactual assemblages to provide key dating material that allowed for subsequent phasing of the amphorae. This timeline showed a strong peak in the middle of the first century AD in almost all products, with amphorae from the Eastern Mediterranean peaking between AD 40–50, products from Italy peaking from AD 50–70, products from North Africa from AD 310–345, the Iberian Peninsula from AD 30–40, and Gaul between AD 50–70 (Fig 9a). Apart from North Africa, most amphorae-borne products consumed at Augst were imported during the first century AD, and far smaller quantities arrived especially after AD 130. Except for material from North Africa and, to a lesser degree, the Iberian Peninsula, very little material arrived in Augst after ca. AD 280. When viewed by contents (Fig 9b), Wine, olive oil, and fish sauce showed very similar patterns of a rapid peak in the middle of the first century AD, followed by significant decline after AD 100. Fish sauce declined most rapidly, becoming negligible by AD 150. Olive oil was consumed in higher quantities than fish sauce in the second and third centuries, but at far lower levels than those seen in the first century. Wine still has a significant presence over the second and third centuries, higher than olive oil, but also far below the levels of the first century. Other products like olives, fruit, and unknowns show far lower levels but a similar chronological pattern, peaking in the mid first century and then declining.

Our own investigation of the material from Augst was able to produce similar graphs for the Augst assemblages using the type dates (which indicate production, context-specific dates are not included in the RAAD database) based on both contents and area of production origin but using the probabilistic aoristic method to calculate the sum of frequency. We were not able to include contextual or phased data within our database, and so here our methods diverge from the original publication. When viewed by origin (Fig 9c), we see a rapid first century AD peak in material from the Iberian Peninsula and, slightly later, Gaul. Iberian material then makes gradual steps down until about AD 280, when it plummets. Gallic material drops more quickly into the second century AD, and then also disappears abruptly by AD 300. The other regions barely feature on this graph, though a first century AD prominence is still visible for Eastern and Italian material. When viewed by contents (Fig 9d), wine shows the earliest, though slow, rise before peaking in the latter first century AD, while fish sauce shows a much more concentrated chronology from roughly AD 1–150, with a peak after AD 50. Olive oil presents the regularly seen table-shaped plateau, with a remarkable rise after AD 1, a level frequency until AD 280, and then a remarkable drop into the later Roman periods. Fig 9e shows the RAAD type number count per year according to each dating method and shows the changes in amphora form diversity through time at this single site. This pattern demonstrates that Augst had the widest availability of amphora types in the later first century AD, and this diversity declined thereafter, with a much more limited form repertoire after AD 200.

### Case study 4: Settlement types

The RAAD database includes information on the types of site present in the dataset (21). Site types are categorized as either military sites (n = 41), civilian settlements (n = 30), villas (n = 6), or oppida (n = 2). The oppida site count is too low to be statistically relevant, so we have removed these from this case study analysis. Some of these sites contain areas that may fit into multiple categories, as they are not all mutually exclusive. We have paid close attention to where on the sites the amphora came from, however, so we make distinctions between the military site of Hofheim Steinkastell (RAAD site 29) and Hofheim Vicus (RAAD site 30). Augst presents a slightly different story, where amphorae from the civilian settlement of Augusta Raurica was published together with the amphorae from the late Roman fortification (which included civilians) of Kaiseraugst [16–18]. To account for this double site publication, we added a fourth category specific to this site, labeled on Fig 8 as “settlement/military (Augst)”: The high number of military sites in the area reflects the Roman reality of life on a heavily fortified frontier, but also the research agendas of archaeologists in the region for the better part of two centuries. These categories of sites consumed amphorae in different ways, and so we also investigated here how patterns of amphora consumption changed over time between the different site types.

**Fig 10:**
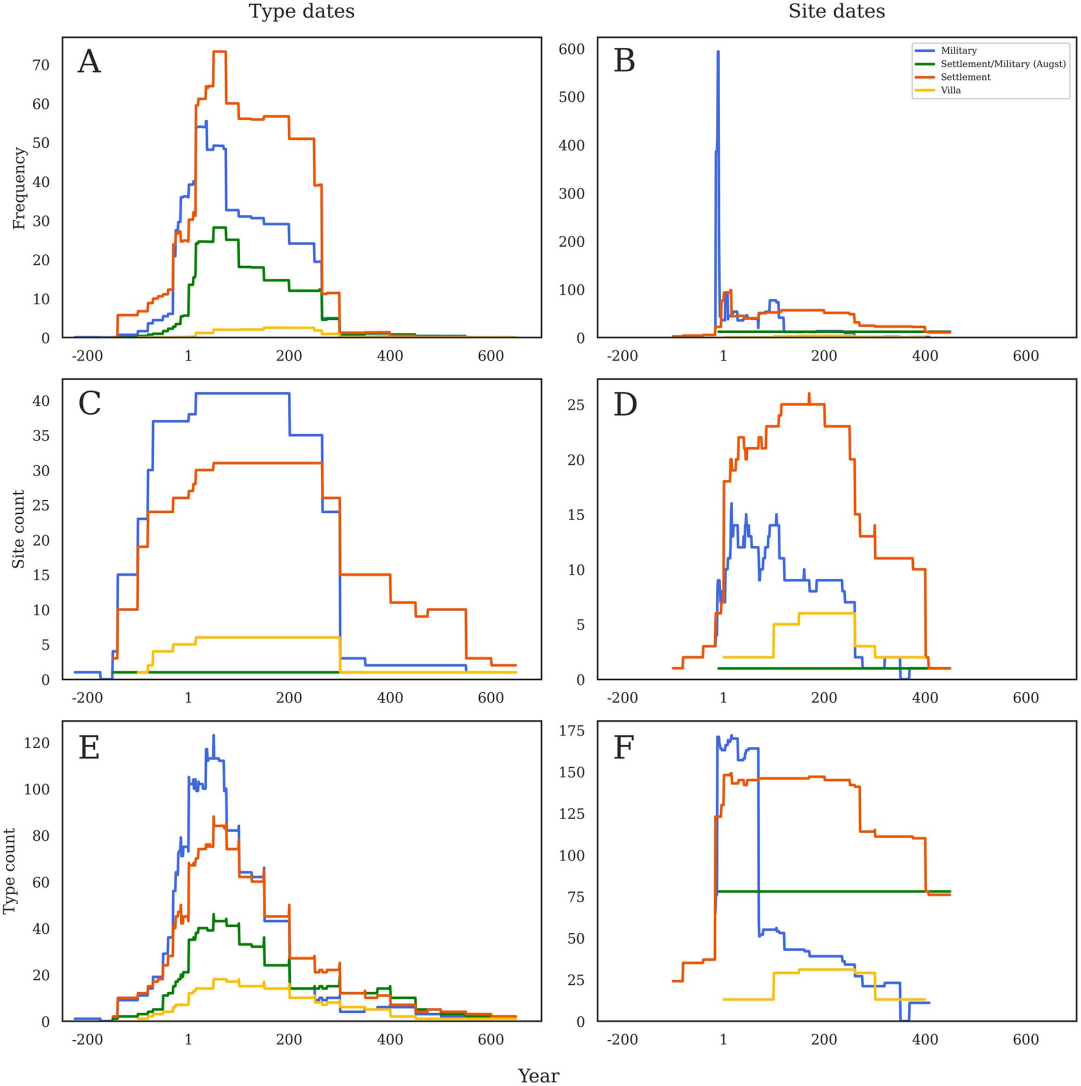
a-f. Settlement types. Comparison of four main settlement types across the three different methods of analysis according to production dates (left) and consumption dates (right). Note that Y axes are not constant.

Fig 10a shows the sum of frequency per site type per year according to typological production chronologies. Here we see sharp increases across military and civilian sites in the late first century BC, with peaks in the first century AD. Military sites have the highest frequency by about AD 25, and civilian sites peak slightly later between about AD 50–75. The military sites then drop off by AD 100, and continue to decline until about AD 265, when the frequency drops near zero. Civilian sites show a much longer plateau after their peak, with higher frequencies present until the middle of the third century AD, before also dropping to almost zero by AD 300. Augst closely mimics the civilian pattern, though with lower frequency. Villa sites have a low frequency that reaches its peak around AD 200, though we should keep in mind the low site count.

Fig 10b shows the sum of frequency per site type per year according to site dates. A dramatic peak in frequency of amphorae consumption on military sites in the late first century BC drowns out the detail of much of the rest of this graph. This sharp spike falls immediately at the start of the first century AD, and then both military and civilian sites show a much lower frequency over the rest of the period. Civilian sites still show a higher frequency for longer, though the military sites show a higher frequency at the start of the second century AD, before essentially disappearing thereafter.

Fig 10c shows the number of sites per year per site type according to amphora production dates. Both military and civilian sites show origins in the mid second century BC, a date clearly far too early to fit with historical reality. More military sites are present in the database until ca. AD 300, with fewer civilian sites across this same period. Civilian sites continue, however, into the late Roman period and beyond whereas many of the military sites disappear in the later period. Villas show a similar pattern, though with lower numbers, and Augst barely registers with this method.

Fig 10d shows the count of sites per year per site type according to site occupation chronology, which shows a much more historically accurate timeline. Here, both civilian and military sites raise in number from the late first century BC, with civilian settlements continuing to increase until ca. AD 200, and the number of military sites rising and falling over the first and second centuries. Settlements again last much longer, and military sites are very rare after AD 265. Amphora-consuming villas are most numerous in the third century, and Augst only represents one site, so stays constant at one the entire time.

Fig 10e shows the RAAD type number count per site type per year according to amphora production dates. With this method, military sites show the highest diversity of forms from the late first century BC until AD 200, with a peak in the middle of the first century AD. Civilian sites show a similar shape but with lower numbers, peaking in the mid first century AD and then tapering to AD 200 where they then overtake military sites for a brief period with higher form diversity. Augst shows a similar pattern to civilian sites, and villas show a less dramatic curve, with a peak in the late first century AD.

Fig 10f shows RAAD type number count per site type per year according to site occupation chronology and shows a significantly different set of patterns than Fig 9e. Military sites show a fast and early peak in diversity, beginning at the very end of the first century BC and lasting until about AD 75, whereafter it declines and fewer and fewer forms can be found in forts up until about AD 350. Civilian sites have a less dramatic rise but maintain a long plateau of form diversity from about AD 1 until AD 400. Villas again show a later peak in the third century, and Augst is again a flat line as it is only a single site (though with relatively high diversity).

### Case study 5: Augustan period sites

**Fig 11:**
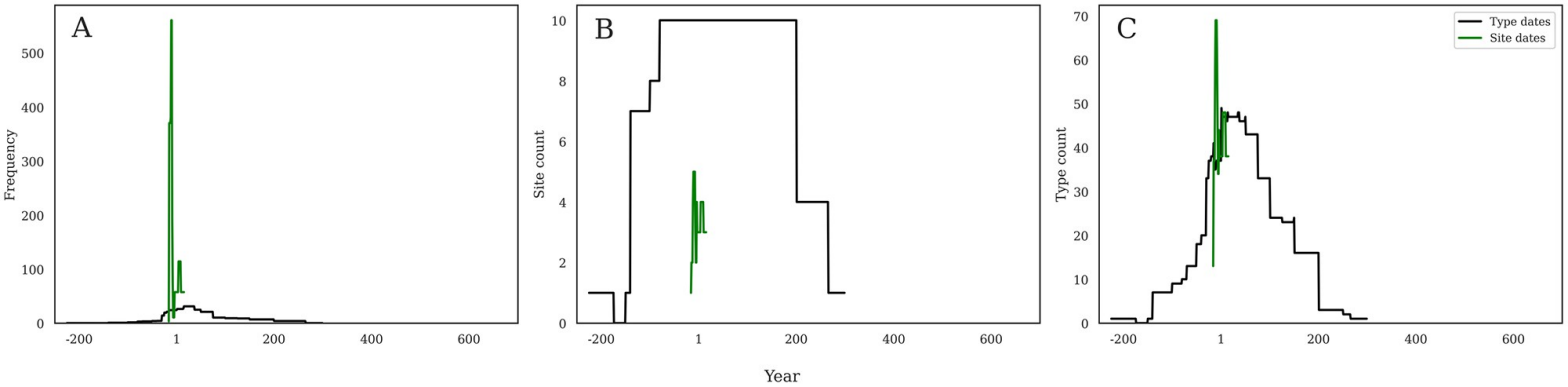
a-c. Augustan-period sites. Comparison of Augustan-period sites across three different methods of analysis according to production dates (black) and consumption dates (green). Note that Y axes are not constant.

The dramatic spike in frequency evident in Augustan period sites is worth further exploration. We separated out the ten assemblages that date specifically to the period of Augustan conquest in Germany, roughly 19 BC—AD 9: Anreppen (RAAD site 5), Dangstetten (RAAD site 21), Höchst (RAAD site 28), Neuss Camps 1–4 (RAAD sites 41–44), Oberaden (RAAD site 51), Rödgen (RAAD site 58), and Waldgirmes (RAAD site 75), and compared their assemblages between the two different dating methods used in this paper. Fig 11a compares the frequency per year per dating method for this group of sites and shows a substantial difference between amphora production chronology and site occupation chronology. When these sites are viewed by production chronology, they show an incredibly wide temporal range that peaks in the first half of the first century AD. This is problematic since all of these sites were only occupied for a very brief period at the end of the first century BC and start of the first century AD, and this historical reality is much more clearly reflected when using the site chronology dating method. When considered in this light, we see the significant spike in frequency already observed in Fig 10b above now singled out as the result of this small selection of sites with narrow date ranges. Fig 11b shows the count of sites per year per dating method, and again the production chronology produces an unrealistic timeline of site occupation that stretches from the second century BC well past AD 200. The site occupation dates, however, show again a much narrower constriction of the timeline to reflect the Augustan campaigns. Fig 11c shows the RAAD type number count per dating type per year for Augustan sites, with the production dates again showing a 400-year chronology for sites only occupied across a roughly 25-year span. Here again the site occupation dates more realistically constrain our dataset and show a more useful picture of the diversity of amphorae being consumed on these Augustan period sites within a very short and tightly bounded period.

## Discussion

Our analytical methods have thus revealed a series of chronological patterns in the consumption of amphorae-borne products along the German frontier over the course of more than five centuries. While there is no single pattern consistent between these analytical methods and quantifications, there are enough similarities to draw new and important conclusions about the economic conditions of the Roman world that led to the creation of this archaeological record, as well as modern archaeological conditions that led to its publication.

### ‘Characterful data’

As our analysis is directly dependent on published data, the choices that went into those publications leading to heterogeneity of the dataset directly affect our outcomes. It is thus worth highlighting several places where modern scholarly practice has clearly influenced the shape of the amphora data patterns we have explored in this paper. Four cases are especially obvious: the dates of sites that have been excavated, the resolution of the ceramic data published after excavation, the date ranges created by scholars to bracket production chronologies of individual forms, and finally, quantification methods.

The database contains a large number of sites that are discretely dated to the Augustan period, especially military sites linked to the earliest military campaigns in the region (see Fig 10). The substantial spike in sum of frequency by site date (see Figs 3a, 10b and 11a–11c) exactly during this period is indicative of this large number of sites with very specific date ranges. This early concentration of sites also contributes to the rapid rise of RAAD type number count per year dated by site dates (see Fig 3c). Aside from these very specifically dated sites, there are also those that have site chronologies that stretch for three or four hundred years, and thus result in a long and largely undifferentiated plateau when using site dates to date amphorae, again especially visible in summed frequency (Fig 3a) and RAAD type count (Fig 3c). These dating particularities do not so readily affect the count of sites per year method, as this does not count amphorae.

The resolution of data published from these sites also has a strong influence on the patterns shown here. First, it is clear that recent publications such as Xanten (RAAD site 79) and Nijmegen Kops Plateau (RAAD site 50) [29,49] present a larger diversity of amphorae than older studies, most probably attributable to both changing methods of excavation and study, as well as wider improvements in ceramic typology. Older publications often present especially the most common forms like Dressel 2–4 (RAAD form 135), Dressel 20 (RAAD form 196), or Gauloise 4 (RAAD form 52), but it is left unstated how many were unidentified or existed in only single examples. Thus, we have a situation in which the most common forms are most common because they are most recognizable and have the longest history of study, whereas other, especially locally produced material, is increasingly playing a larger role in modern studies. Thus, when our analyses focus on either production origin or contents, we are limited in what publications present, and there is no doubt that the increasingly diverse assemblages being published in the region may skew some of the patterns highlighted here.

**Fig 12:**
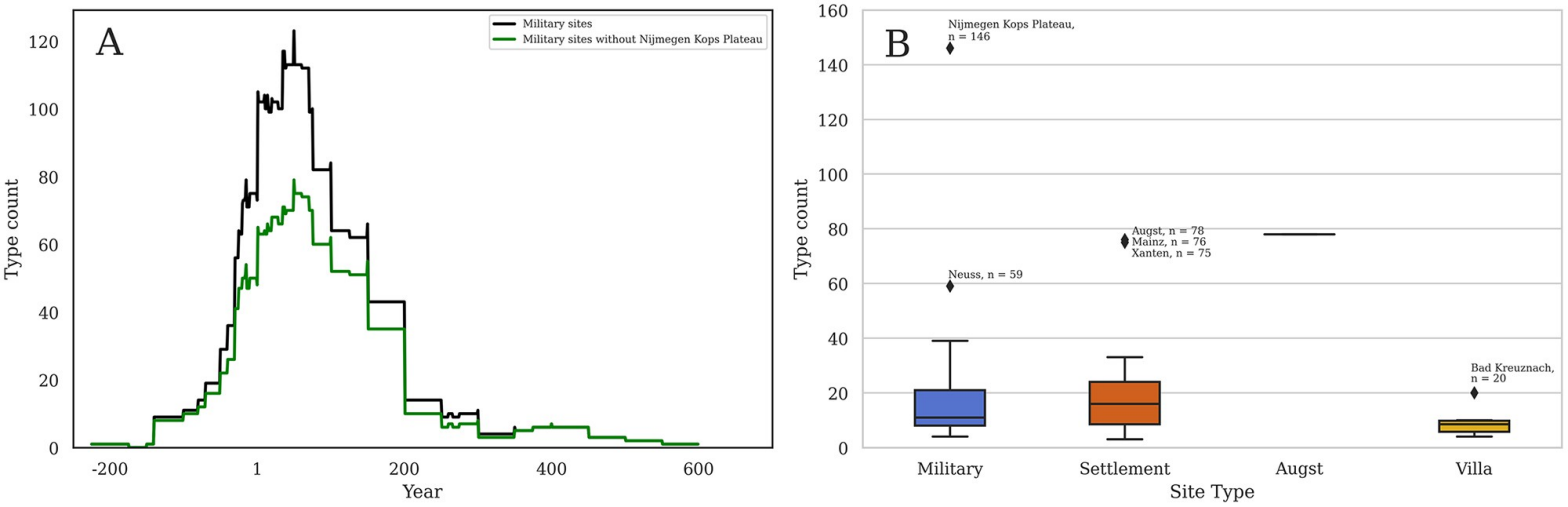
a-b: Military diversity. (a) Comparison of RAAD type number per site type per year according to type dates between all military sites excluding Nijmegen Kops Plateau (black) and Nijmegen Kops Plateau only (green). (b) Box and whisker plot of diversity of forms across settlement types with outliers marked.

Our methods and analyses outlined in this paper highlight the influence of this past research, especially evident with an assemblage such as Nijmegen Kops Plateau (RAAD site 50) where researchers identified and published 146 individual forms [49]. Fig 12a compares the RAAD type number per site type per year according to type dates of all military sites excluding Nijmegen Kops Plateau (black) and Nijmegen Kops Plateau only (green), clearly demonstrating the influence of this single site on shaping the data trend seen in Fig 9e. When one considers that the average number of individual forms on Roman military sites contained in the database is 17.8 including Nijmegen Kops Plateau or 14.6 excluding Nijmegen Kops Plateau (Fig 12b), this disparity is especially apparent. The detailed work at the civilian settlements of Augst (RAAD site 9) [16–18], Mainz (RAAD site 38) [19], and Xanten (RAAD site 79) [29] also produced a high number of individual forms with 78, 76, and 75 different forms respectively, all outliers compared to the average settlement diversity of 21.3 different forms. All these outliers are major sites in the area, either legionary bases or urban administrative capitals, so we might expect higher diversity regardless, though it is noteworthy that comparable sites such as Vetera I (RAAD site 71) or Avenches (RAAD site 10) show lower diversity. It is notable that each of these sites with high numbers of different forms are published in monograph-length investigations of the material, and the disparity in detail is apparent.

It must be remembered that all the dates used here, whether for amphorae forms, sites chronologies, or historical events are, to some extent, artificial brackets that have been created and reified by archaeological and historical research. The Augustan period sites are one part of this, but so too is the prominent end date of AD 260, the traditional date of the abandonment of the outer German limes as well as all Roman territory across the Rhine in Baden-Wurttemberg. Amphorae production chronologies are often given in 25-, 50-, or 100-year increments, and necessarily are estimates. The effect of this chronological blocking is perhaps most clearly seen in the calculation of RAAD type number count per year by type date (Fig 3c), as roughly 25-year steps are visible in the decline after ca. AD 50–these steps are formed by the dates used for amphora chronologies, and thus probably blur the historical reality to variable extents. It is therefore crucial to emphasize that our interpretation of the data patterns should focus exclusively on the larger trends, since small fluctuations or changes on rounded dates are creations of this practice. Monte Carlo methods could be used in future research (Crema 2012) as a probabilistic approach that more explicitly represents the need to focus our quantitative exploration on major trends.

Finally, when considering the quantification method, we can see the influence of particular researchers on the representation of data. Ulrike Ehmig’s extensive and invaluable work on amphorae in Germany is the most obvious case [19,28,50,51]. She uses the maximum number of individuals (MAX) method throughout her work, and it is obvious in Fig 4d especially that this method shapes the dataset in a specific way. The sites that Ehmig has published tend to cluster in two main chronological periods–the Augustan period at Dangstetten (RAAD site 21) and Waldgirmes (RAAD site 75), and then the second and third century sites in the hinterland of Mainz. Thus, the shape of data quantified by her methods stands apart from the others, and especially peaks in the Augustan and later second/third centuries when the other quantification methods show decline. This later peak is not so much an artifact of actual economic processes, but rather that most of the sites from this period are quantified by one scholar using a particular method. This important fact is obscured in the process of data synthesis but is very apparent when exploring published evidence with our quantification methods.

### Comparison of dating methods

We used two methods of establishing chronologies within our data: the first utilizing the ‘production chronologies’ of individual amphorae forms based on when the earliest and latest versions of forms are known, the second using the ‘consumption chronologies’ of individual sites where the start and end dates of occupation are known. We used both methods to account for some of the characterful ambiguity present in the many assemblages that are not published with phasing, as well as to fill in holes in knowledge such as when specific amphorae forms were produced and consumed. The strong contrast revealed by our results between the data patterns when using either typological or site dates should clearly caution against the exclusive use of typological dates of amphorae for interpreting when the foodstuffs contained in them were consumed. We demonstrate here that amphora production and consumption behaviour result in quantitatively different temporal patterning in published material culture, and we therefore treat them as separate but intertwined phenomena.

Production chronologies have the potential to cover very large date ranges that sometimes extend outside the period of Roman rule in the German area. We pointed out above that Italian and Eastern Mediterranean wine amphorae especially illustrate this point (Fig 7), and they contribute to creating a chronology that does not fully represent the economic history of the German frontier. On the other hand, consumption chronologies are constrained to the periods of site occupation where amphorae are found and are therefore more representative of local patterns of deposition. However, because some of these site chronologies are very long, they also hold the potential to create long-lasting plateaus where there is little differentiation through time, as outlined in the previous section.

The most problematic aspect of either method are amphorae forms that have a long production chronology but only a very specific chronology of consumption in Germany. Italian wine emphasizes this point (Fig 7a and 7b), as already stated, and so here the site dates are very helpful to constrain our estimates of chronology. To turn this around, this analysis also provides a window into the marketing history of wine from an Italian perspective: the long history of wine production in Italy only very briefly turned its attention to consumers in the German market, but this in no way implies a cessation of Italian production or its consumption elsewhere.

On the other hand, our methods do work well when amphorae production chronologies fall entirely within the period of Roman rule in the German region. This is especially evident in both Gallic wine and Spanish olive oil (Figs 7 and 8; see below on both) imported in Dressel 20 (RAAd form 196) and Gauloise 4 (RAAD form 52) containers. These chronologies are consistently similar between our two dating methods.

Thus, neither method is perfect on its own, but both used in conjunction help to narrow and clarify the chronologies of amphorae importation, trade, and consumption within the region. With these caveats in mind, some chronological patterns are now clear. First, most amphorae contained in this database were produced and consumed from the late first century BC and throughout the first century AD. The second century AD saw a marked drop in all products from all production regions, except for locally produced amphorae that may have, at least sometimes, contained wine (see Fig 7a–7d). The late Roman period, from ca. AD 260 onwards, shows very little evidence of amphorae-borne products in this region.

### Regional product consumption and long-distance trade connections

Our analyses reveal a highly heterogeneous history of long-distance trade between the German frontier and many regions of the Roman Empire. We divided our dataset into six different regions of origin: Gaul, Germania, the Iberian Peninsula, the Eastern Mediterranean, North Africa, and Italy, and the German region imported goods from all these regions throughout its history. The widest variety of products from across the Empire arrived at the German frontier in the late first century BC and the first century AD (Fig 5e and 5f), reflecting a period of widespread connections as well as a highly diverse availability of products in Germany. Woolf has characterized this rapid increase in commercial products newly available in the region as a “consumer revolution [52]” in Roman Gaul in which the material culture of the region was “richer and more various [52]” than its Iron Age predecessors. This period of simultaneous Empire-wide connections was, however, relatively brief, and it is especially clear that connections with Italy and the Eastern Mediterranean were early phenomena that came to be replaced by dominant connections with especially southern Spain and southern Gaul over the course of the first century AD (Fig 5a and 5b). Thus, the early diversity gave way to much more sustained connections with two primary regions, and material that filtered in from beyond these can be considered as rarities. German products, mainly produced in and around the Rhineland, begin to appear in serious quantities by about AD 100, and reach higher levels of frequency (Fig 5a and 5b) and distribution (Fig 5c and 5d) than many other origins (except Gaul and the Iberian Peninsula) from this point until the middle of the third century.

This gradually constricting market demonstrates a high degree of import replacement that is visible along the German frontier in a variety of material categories, including especially terra sigillata pottery and glass [15]. An initially wide variety of products that drew from all over the Empire narrowed to very specific connections with two regions, southern Spain and southern France, to produce two products which had no precedent in Germany: olive oil and wine. The productive industries in these two regions developed hand-in-hand, as Gallic wine producers recognized the opportunity to focus on a different product while capitalizing on the same transportation infrastructure. As time went on, even these dominant connections declined, and it is possible that the increase in German amphorae over the second century AD is related to new agricultural enterprises invested in the production of vegetal oils and wine, now carried in locally produced containers.

It is also clear that most of these connections, even with the Iberian Peninsula and Gaul, ended by the middle of the third century AD. The number of later Roman amphorae found on the German frontier is so comparatively small that it is very difficult to draw many conclusions other than some rare imports still arrived, potentially foreign and exotic wines of desired vintage from the Eastern Mediterranean [20] as well as North Africa (Fig 7a–7d).

There are two very different ways to interpret these changing connections: economic failure demonstrated by an inability to maintain long-distance trade or economic success demonstrated by an increasingly localized and contained economy that did not need to rely upon outside producers for its foodstuffs. These two scenarios are explored in more depth in the following sections.

### Changes in products imported

The four different products that we studied (olive oil, wine, fish sauce, and fruit) arrived in very different patterns through time (Fig 6a–6f). Wine had the longest history of consumption, predating Roman conquest and continuing straight through the end. Olive oil shows a mostly constrained importation between AD 1–260, with very little evidence of later importation. Fish sauce was particularly popular early in the region’s history, with most of its imports occurring in the first century AD. Fruit is also a short-lived product and only arrived in small quantities in the first and early second centuries AD.

Wine and olive oil dominate assemblages in the region, and the other products are only ever ancillary to these two main imported staples. It is highly possible that Mediterranean fish sauce was not well received in local cuisines, but it is also probable that more localized fishing industries and subsequent production of fish sauces took over this market, possibly in the face of declining Mediterranean production [53]. These local fish products were almost certainly shipped in barrels, as no ceramic containers are known from this industry. Patterns in wine and oil consumption are explored in more detail below.

### Wine

Wine was imported to Germany from all over the Empire throughout the Roman period and, while most of it arrived in the first two and a half centuries AD, there was some sustained importation later, especially from the Eastern Mediterranean (Fig 7). Gallic wine was by far the most common, however, regardless of method of analysis, quantification, or dating. This dominance begins in the middle of the first century AD and lasts through the third century, reflecting the same basic chronology of the majority of production in southern France, where agricultural estates began to specialize their production in wine to meet market demands from Germany and other regions [54]. If the amphorae produced in Germany contained wine, as we hypothesized above, then Gallic wine gets some competition in the later second century AD, as these containers become more common. Paradoxically, however, archaeological evidence for wine production facilities in Germany only appears in the later third century AD [55,56] in the Moselle and middle Rhine valleys. If amphorae like the Niederbieber 74/75 did carry wine, then we do not yet know where it was grown in the region.

It is also possible that these German amphorae, especially the Dressel 20 *similis* (RAAD form 107), contained beer [19,28,57,58]. If this were the case, then the increase in locally produced containers may not reflect a local wine industry striving to cut into foreign trade, but rather a local taste preference that developed alongside wine. Like the rest of the dataset, wine amphorae abruptly declined during the third century AD. The abrupt end to both Gallic and Germanic amphorae visible in the archaeological record is probably not indicative, though, of a cessation of wine consumption, but rather a change in containers from amphorae to barrels, discussed more below.

### Olive oil

Olive oil was imported from three regions: southern Spain (included here within the Iberian Peninsula), Adriatic Italy, and North Africa. The importance of Baetican olive oil from southern Spain to the German market cannot be overstated, as it is by far the dominant product not just within olive oil containers, but in the entire regional amphorae assemblage. Unlike fish products, wine, and beer, the German frontier could not produce olives, being located well outside the Mediterranean-centered zone of olive cultivation, and so was entirely reliant on Mediterranean imports for this product. Much like the wine industry in southern Gaul, Baetican olive growers made a decision to specialize their efforts in the production of oil to meet these demands, and this is evident in the archaeological record of the region where dozens of estates and bottling facilities are known [13,59,60].

Roman occupation of Germany began at almost the same time as Spanish olive oil production, and the links between the two regions have been long explored [13,59,60]. Italian and North African olive oil was never able to contend with the sheer quantity of Spanish olive oil and the Spanish dominance revealed by our analyses is remarkably consistent across methods: a significant plateau that stretches for two and a half centuries (Fig 8). Because this main pattern in both production and consumption dates is comparable (i.e. a strong focus on the first three centuries AD, Spanish oil is probably the clearest demonstration of the methods used in this paper, as these patterns are not unduly influenced by dating problems or, indeed, many other of the “characterful” issues that arise in these data.

That said, olive oil does disappear from the record by the end of the third century AD. This corresponds to the end of production of the Dressel 20 (RAAD form 196) in southern Spain around AD 260, and the appearance of the new, smaller container of the Dressel 23 (RAAD form 198). These late Spanish oil containers are barely known in Germany, with only a handful present in the later centuries of Roman rule. Thus, we see an end of the importation of Spanish oil in the late third century, the reasons for which are not entirely clear. Since Spanish olive oil was still being shipped elsewhere in amphorae, it seems unlikely that there was any kind of switch to barrels for transportation north.

### The role of barrels

We notice a strong decline in the frequency, distribution, and diversity of amphorae from the third century AD onwards (Fig 3), but does that decline reflect an end to the importation of amphora-borne foodstuffs? While the presence of amphorae is good evidence for the presence of the commodities that they carried, the absence of amphorae does not necessarily indicate the absence of that same commodity (Fig 6). The role of barrels in the long-distance transportation of bulk commodities has been extensively discussed [61–63], and the northern provinces do, in fact, preserve extensive evidence for barrels in anaerobic archaeological conditions. The barrels known from the German frontier overwhelmingly date to the early Roman period, a fact almost entirely dependent on the method of well construction that reused barrels as cladding [15]. There is no apparent late Roman barrel prominence in these data, though of course the absence of evidence is not evidence of absence, and barrels occur on Roman monuments and tombstones throughout the region, as at Neumagen, Germany and Colijnsplaat, Netherlands in the second and third centuries AD.

The use of barrels in the movement of bulk liquid cargoes is believed to be both cheaper and more resource-appropriate for certain producers and transporters, as timber resources are more prominent in some locations than claybeds, and barrel manufacturing does not require capital investment in, for example, kilns. It is perhaps the case that barrels more easily fit onto the flat-bottomed barges that were most commonly used to transport goods on the rivers of Roman Europe because of their flat bases, but there were also flat-bottomed amphorae in use in the same region for the same purpose. Barrels are not universally appropriate for carrying the same commodities as amphorae, due mainly to their porosity. Wine and salted fish products could be shipped in barrels and, in Gaul and Germany at least, wine was probably the most commonly shipped product. Excavations at Vindolanda near Hadrian’s Wall in Northern Britain have demonstrated the use of barrels for bulk wine importation, estimating that 79% or 11,001 liters of the products typically thought to be transported in amphorae actually arrived at Vindolanda in barrels, mostly from Gaul in the late first and early second century AD [64,65]. The authors argue that this wine transported in barrels would probably have been of low quality, meant for the troops, while officers would have had access to the higher quality wines in amphorae. The extent to which we should extrapolate this particular site-based situation is entirely unclear, though it highlights the possible difference in volume between the two container types.

On the German frontier, it is very clear that there were barrels in use, probably extensively, and we cannot accurately quantify their impact. Thus, the dramatic reduction of wine amphorae after the third century (Fig 7) may well reflect a change in containers. The wineries that develop along the Moselle River typically include extensive open rooms interpreted as cellars for the storage of wine barrels, and no amphora production is known locally [55]. Our results instead show changes in the patterns of consumption of wines that were still shipped in amphorae, and it is highly unlikely that prized vintages from distant regions were transferred into wooden containers prior to sale, even in later periods. It is the Gallic and German wine that probably made the change (their data patterns stopping quite abruptly around the middle third century AD; (Fig 7), taking advantage of local wood sources that were much more prominent than those in the Mediterranean. Locally produced fish sauce may also have been stored in barrels [53] but, as noted above, olive oil probably was not.

### Military and civilian consumers

The role of the military is a central concern in understanding the motivations behind economic connections in a frontier zone of the Roman Empire, and much has been said about the economic weight of the Roman military in attracting food products over long distances [11–13,66,67]. We explicitly explored and compared trends in consumption between military and civilian sites and found that civilian settlements had longer-sustained summed frequencies regardless of dating method (Fig 10a and 10b), a much more sustained presence of sites with amphorae through time, especially when dated by site occupation date (Fig 10d), and also had a more diverse assortment of products to consume than military sites after the Augustan period (Fig 10e and 10f), especially when the outlier site of Nijmegen Kops Plateau is removed from the military calculations (Fig 12a). All of these patterns call into question the long-held tradition that soldiers–not civilians–attracted goods to frontier regions of the Empire (especially [12]), and demonstrate that civilian centers actually carried significant economic weight on the edges of Empire, and had equal, if not better, access to amphora-borne foodstuffs (see also [15,20]). It is also important to note that this access at civilian settlements was much more sustained through time, again highlighting differences between the two consumer groups.

This observation is significant in Roman economic history because it poses a substantial problem for economic models that prefer an economically fruitful and successful Mediterranean core that then subsidized other regions of the Empire, most notably the city of Rome and frontier zones, through tax redistribution [11,68]. While many have criticized these models as too coarse and impressionistic [1], few have yet to explore the implications of material-based study for understanding frontier market integrations [15]. This paper demonstrates through multiple methods that military sites were able to procure and consume amphora-borne commodities, but civilian sites were able to procure and consume a higher number and larger diversity of products at the same time. Thus, the economic and market force of civilian settlements must be considered in frontier zones, and they strongly speak against the notion of economically inferior regions that were reliant upon state subsidies in foodstuffs.

### Site-based investigations vs the region

The methods that we have explored here have been shown to be effective at examining the macro-scale economic patterns of amphorae consumption, but we have also highlighted the difficulties in using these methods to focus on singular sites by focusing on Augst (RAAD site 9) as a case study (Fig 9). Our methods are best aimed at synthetic analysis of multiple data points, and some necessarily require multi-site input (i.e. count of sites Fig 9f and 9g), and the method of using site-based consumption dates of course does not work at the individual site scale (Fig 9e). Thus, we intend the methods discussed in this paper to be applicable to other regional studies that, like here, build upon excavated and published material that contains some level of uncertainty.

## Conclusions

In this paper we investigated many different aspects of the regional amphora assemblages of 79 sites from the German frontier. This scale of investigation for amphorae has never been attempted in Roman studies. Previous synthetic work has been more limited by issues of uncertainty in dating and typology, and most good quantitative, long-term analysis is based on single sites [16–18] with well-phased ceramic assemblages. The methods we have used here explicitly address this ambiguity and heterogeneity, demonstrating new ways to quantify and date ceramic assemblages at a broad, synthetic scale. Moreover, the analysis and visualization methods we used are transparent and reproducible and can be applied in other case studies of the Roman Empire or beyond in the future. This has enabled us to reveal completely novel and detailed chronological patterns of previously published data at unprecedented scales for a region not often included in economic discussions that are more often Mediterranean-focused.

The rich archaeological record of the German region, as well its neighboring territories of northern Europe, however, provide ample material for such studies, and these regions should be included in economic history more often. Our focus on a frontier region has allowed for careful analysis of economic patterns on the edge of the Roman world, in a social context where economic activity is most often conceived by scholars as a command economy that was State-organized and isolated from the wealthier Mediterranean [11,14]. We have demonstrated here that such perceptions are false and, in fact, that the German frontier was closely connected to far-flung regions throughout its existence. By investigating long-distance trade connections, this work also reveals elements of production strategies in widely different regions, stretching from the Iberian Peninsula to the Levant, and from North Africa to the Rhineland. Aside from merely demonstrating these connections that were otherwise obvious in any archaeological publication, we have also revealed their highly detailed dynamism through time in both scale and duration. We show an intense period of Empire-wide integration early on, in which the German frontier consumed products from the entire Empire. The subsequent reduction of these connections that we here reveal represent changes in these trade connections, especially emphasizing local import-replacement in which local producers seize upon the opportunity to meet the demands of their markets at lower costs for consumers. The German frontier increasingly came to rely on more localized products, first in the Western Empire and then very locally. Thus, reduction of trade connections is not here interpreted necessarily as economic decline, but rather as local success that beat out foreign competitors. This data-driven chronological narrative of Empire-wide integration and disintegration is novel, and has very rarely been explored in the Roman world, especially outside the Mediterranean (though see [69**]** for an Hispanic case study and [70] for comparative study of amphora assemblages in Mediterranean ports).

The result of these innovations in synthesis and analysis is a comprehensive view of the market for amphorae-borne food commodities along the German frontier that reveals extensive patterns in long-distance trade connections with other regions of the Roman Empire, as well as changing patterns of taste and preference amongst local consumers. We reveal chronological and geographical pulsations of market connections that were highly heterogeneous and dynamic. These connections are important not only for understanding patterns of consumption in Germany, but also for understanding the regional productive economies of the Roman world–producers knew what regions provided demand, and they matched this demand with output.
